# Multiple cation insertion into a polyaromatic hydrocarbon guided by data and computation†

**DOI:** 10.1039/d4sc05128a

**Published:** 2024-12-09

**Authors:** Moinak Dutta, Angelos B. Canaj, Tilen Knaflič, Christopher M. Collins, Troy D. Manning, Hongjun Niu, Luke M. Daniels, Aikaterini Vriza, Luke A. Johnson, Bhupendra P. Mali, Yuri Tanuma, T. Wesley Surta, John B. Claridge, Neil G. Berry, Denis Arčon, Matthew S. Dyer, Matthew J. Rosseinsky

**Affiliations:** a Materials Innovation Factory, Department of Chemistry, University of Liverpool 51 Oxford Street L7 3NY Liverpool UK rossein@liverpool.ac.uk; b Condensed Matter Physics Department, Jožef Stefan Institute Jamova 39 SI-1000 Ljubljana Slovenia; c Institute for the Protection of Cultural Heritage of Slovenia, Research Institute Poljanska cesta 40 SI-1000 Ljubljana Slovenia; d Faculty of Mathematics and Physics, University of Ljubljana Jadranska 19 SI-1000 Ljubljana Slovenia

## Abstract

We report the synthesis, structural characterization and magnetic properties of K_3_coronene, and demonstrate a computational screening workflow designed to accelerate the discovery of metal intercalated polycyclic aromatic hydrocarbon (PAH), a class of materials of interest following reports of superconductivity, but lacking demonstrated and understood characterised material compositions. Coronene is identified as a suitable PAH candidate from a library of PAHs for potassium intercalation by computational screening of their electronic structure and of the void space in their crystal structures, targeting LUMO similarity to C_60_ and the availability of suitable sites to accommodate inserted cations. Convex hull calculations with energies from crystal structure prediction based on ion insertion into the identified void space of coronene suggest that the *x* = 3 composition in K_*x*_coronene is stable at 0 K, reinforcing the suitability of coronone for experimental investigation. Exploration of reaction conditions and compositions revealed that the mild reducing agent KH allows formation of K_3_coronene. The structure of K_3_coronene solved from synchrotron powder X-ray diffraction features extensive reorientation and associated disorder of coronene molecules compared with the parent pristine host. This is driven by K^+^ intercalation and occupation of sites both within and between the coronene stacks that are partially retained from the parent structure. This disruption of the host structure is greater when three cations are inserted per coronene than in reported metal PAH structures where the maximum ratio of cations to PAH is 2. Superconductivity is not observed, contrary to previous reports on K_*x*_coronene. The expected localised moment response of coronene^3−^ is suppressed, which may be associated with the combination of extensive disorder and close coronene^3−^–coronene^3−^ contacts.

## Abstract 2

K_3_coronene is computationally predicted and synthesised. Crystal structure solution reveals a highly disordered structure with evidence of local spin-singlet formation from magnetic property and EPR measurements.

## Graphical abstract

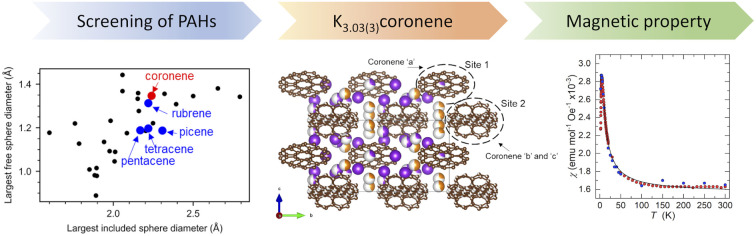

## 1. Introduction

Alkali metal intercalated polycyclic aromatic hydrocarbons (PAHs) have been widely investigated following the reports of superconductivity at critical temperature, *T*_C_ ∼18 K for K_3.3_picene.^1^ Superconductivity was then subsequently reported for phenanthrene-,^2^ chrysene-,^3^ dibenzopentacene-,^4^ triphenylene-^5^ and coronene-based^6^ materials with *T*_C_ as high as 123 K for K-doped *p*-terphenyl.^7^ However, a lack of reproducibility, very small reported superconducting shielding fractions (∼1%) and the absence of detailed structural characterization hinder the progress required to understand the properties of these materials and even led the scientific community to question the claims of superconductivity.^8^

One of the major difficulties in synthesizing and characterizing phase pure alkali metal intercalated PAHs is the complex reactivity of PAHs under reducing conditions^9,10^ and the extreme sensitivity of these intercalated materials to oxygen and moisture. Use of strong reducing agents such as K metal is found to break the C–H *σ* bonds,^8^ generally yielding KH as the major product, along with intercalated amorphous phase(s), instead of or together with any crystalline phase.^9^ Solid-state synthesis using the milder reducing agent KH has been successful in reliably synthesizing and characterizing K^+^ intercalated PAHs, namely, K_2_picene, K_2_pentacene, K_2_rubrene and K_2_tetracene.^9,11,12^ Solution synthesis affords the solvent-free Cs_1_phenanthrene.^10^ However, none of the aforementioned compounds, which are characterized fully, show superconductivity.

The challenges in developing suitable synthesis protocols mean that appropriate selection of PAH targets for intercalation would be invaluable in accelerating the exploration of this chemistry. Pristine picene, pentacene, rubrene and tetracene all adopt herringbone packing with alternating layers having parallel rows of aligned molecules with opposing inclinations (Fig. S1, ESI†). Given these structural similarities between the PAHs where alkali metal intercalation has been demonstrated, alternative PAHs with similar structures (Fig. S1, ESI†) can be identified as potential hosts. We present a combined computational and experimental methodology where PAH candidates are evaluated by considering the porosity in the solid crystalline form of a PAH as a measure of its propensity for the intercalation of metal atoms. This evaluation was combined with assessment of their electronic structures and benchmarked against C_60_, the parent molecule of the A_3_C_60_ (A = alkali metal) superconductors.^13^ Coronene was identified as the most promising candidate, with convex hull calculations based on simple metal insertion model structures suggesting K_3_coronene as the most appropriate target composition. Successful experimental synthesis of K_3_coronene allowed insertion of three alkali cations per PAH to be demonstrated experimentally, and subsequent understanding of the material's magnetic properties.

## 2. Results and discussion

### 2.1. Computationally guided selection of PAH for potassium intercalation

**Fig. 1:**
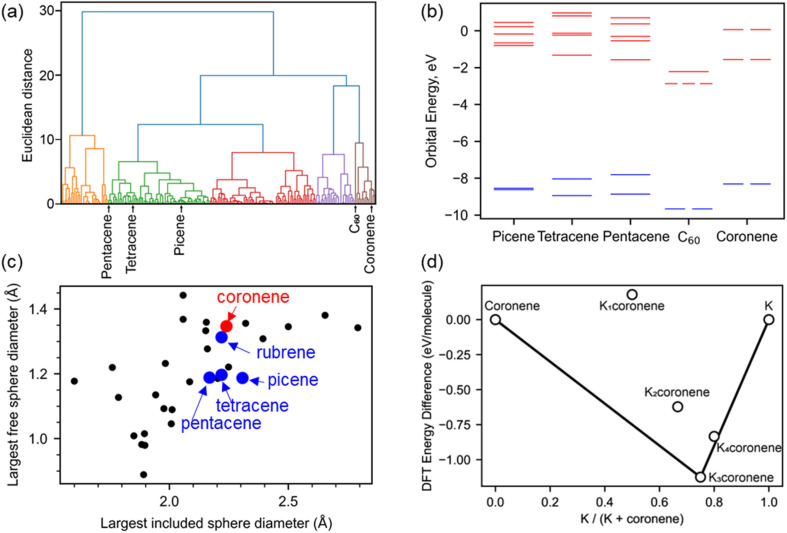
(a) Hierarchical clustering of 195 aromatic PAHs based on their molecular orbital energies from HOMO−1 to LUMO+4. Coronene is clustered with C_60_ and away from picene, pentacene and tetracene. (b) The six molecular orbital energies used for the clustering are plotted, comparing coronene with C_60_, picene, pentacene and tetracene. Coronene and C_60_ have a degenerate lowest unoccupied molecular orbital (LUMO), unlike the other three PAHs compared here. Occupied molecular orbitals are plotted in blue, unoccupied in red (c) porosity analysis of 30 PAHs quantified by finding the diameter of the largest included sphere and free sphere using Zeo++,^14^ C_60_ falls significantly outside of the range for most PAHs, with the largest included sphere diameter of 3.61 Å (*x* co-ordinate) and the largest free sphere diameter of 1.17 Å (*y* co-ordinate), and so is omitted from this plot. (d) Convex hull constructed for K_*x*_coronene with *x* = 1–4, following density functional theory (DFT) calculations on structures (Fig. S2, ESI†) obtained using a simple intercalation approach as described in Computation details, Methods in ESI.† The difference in DFT energy between the intercalated structure and pristine coronene and K is plotted, with the convex hull shown as a solid black line. The most stable composition identified is for *x* = 3 (*i.e.*, K_3_coronene) which lies on the convex hull.

We survey available PAHs to identify suitable candidates for synthesis of intercalated crystalline phases, using criteria of electronic and crystal structure coupled with quantitative prediction of the thermodynamic stability of metal intercalated candidate compositions. Screening for promising PAHs for metal intercalation was done following three criteria: (1) presence of a degenerate lowest occupied molecular orbital (LUMO), based on analogy with the electronic structure of C_60_ and its triply degenerate LUMO (Fig. 1a and b): the orbital degeneracy of the C_60_ LUMO has been identified in both phonon-driven and exotic explanations^15^ of superconductivity in these systems.^16,17^ (2) the presence of void space similar to that in hosts known to successfully form intercalated PAH systems and therefore likely to be able to host metal atoms (Fig. 1c) and (3) quantitative evaluation of the energetic viability of intercalation by DFT relaxation of K_*x*_PAH structures where the cations are located on sites within the void space identified in criterion (2) (Fig. 1d). This is done to enhance confidence in committing the extensive effort likely needed to identify appropriate synthetic conditions to isolate any intercalated material.

In our analysis of 195 PAHs, hierarchical clustering was used to group molecules by similarity of their molecular orbital energies from HOMO−1 to LUMO+4. Coronene was in the same cluster as C_60_ (Fig. 1a), and thus prioritised for further investigation as the A_3_C_60_ (A = alkali metal) phases are confirmed superconductors.^13^ Coronene also displays orbital degeneracy in the LUMO, in contrast to the other PAHs (picene, pentacene, tetracene *etc*.) for which metal intercalation has been unequivocally established previously (Fig. 1b).^18^ Kekulene (C_48_H_24_), in the same cluster as C_60_ and coronene, also shows degenerate LUMO and LUMO+1 orbitals.

In addition to the electronic structure, the crystal structure of a candidate needs to have sufficient void space to be able to accommodate intercalated cations. Porosity screening, as a proxy for void space, of 30 PAHs whose structures are reported in the Cambridge Structural Database (CSD)^19^ was performed by finding the diameter of the largest included sphere and free sphere using Zeo++.^14^ These metrics were used to compare PAH porosity (Fig. 1c and Table S1, ESI†). This evaluation reveals that the porosity in pristine coronene is similar to that of the PAHs demonstrated to intercalate potassium. Kekulene, with a largest free sphere diameter of 2.06 Å, and the largest included sphere diameter of 1.44 Å, is also a suitable candidate for experimental studies but was not considered for further exploration due to not being easily available commercially. Coronene is thus prioritised for study based on both electronic and crystal structure grounds.

**Fig. 2:**
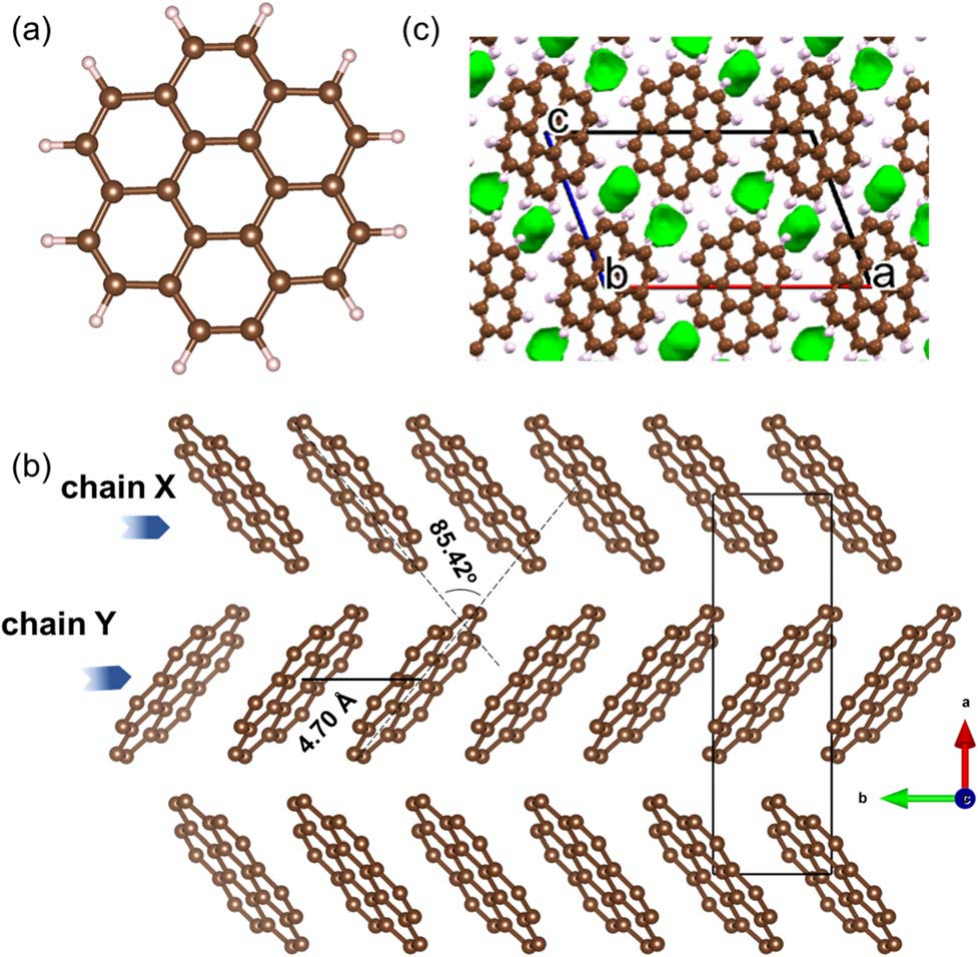
(a) Molecular structure of coronene (C_24_H_12_). Brown and light pink spheres represent carbon and hydrogen atoms, respectively. (b) Herringbone packing of coronene viewed along the crystallographic *c*-direction with hydrogen atoms omitted. (c) Void space in crystalline coronene computed and plotted using Mercury^20^ with a probe radius of 0.65 Å, showing voids lie between the chains.

Coronene (C_24_H_12_), is a PAH that belongs to the sub-class of circumarenes wherein the central arene (in this case benzene) is completely enclosed by another outer ring of fused benzene rings (Fig. 2a). Pristine coronene crystallizes in the monoclinic space group *P*2_1_/*a* with *a* = 16.119(6) Å, *b* = 4.702(4) Å, *c* = 10.102(6) Å and *β* = 110.9(1)°.^21^ Coronene adopts a herringbone packing similar to picene,^9,18^ containing two crystallographically equivalent molecules in the lattice centred at (0, 0, 0) and (½, ½, 0) that are tilted at an interplanar angle (*ω*) of ∼85.42° (Fig. 2b),^18,21^ forming two alternating chains (X and Y) of coronene molecules stacked along the crystallographic *b*-axis that are separated by a distance of 4.70 Å, with voids located between adjacent chains (Fig. 2c).

Density functional theory (DFT) calculations were performed on hypothetical structures of K_*x*_coronene (*x* = 1–4) by first inserting K in the largest existing void spaces in pristine coronene, located using Mercury (Fig. 2c and S3 ESI†) and on the basis of the porosity calculated using Zeo++, and then relaxing the resulting structures. The energies of the structures that resulted from this simple metal intercalation approach were then compared with those of solid crystalline coronene and K to produce the convex hull shown in Fig. 1d. These calculations indicate that K_*x*_coronene (*x* = 2–4) are more stable than isolated coronene and K, with K_3_coronene being on the convex hull and thereby most likely to form (Fig. 1d). The lowest energy structure of K_3_coronene optimized using plane–wave based density functional theory (DFT) as implemented in VASP^22^ (see Computational details in Methods, ESI†) revealed a significant rearrangement of the coronene molecules to accommodate the K^+^ ions. The rearrangement resulted in an increase of the coronene–coronene centre distance to 7.91 Å from 4.70 Å, the angle between two coronene molecules being increased to 101.24° from 85.42° (Fig. S2 and S3, ESI†). The position of K_3_coronene on the convex hull suggests that the energetic cost of the positional and orientational coronene rearrangement can be overcome by the electrostatic energies associated with intercalation to afford the salt.

### 2.2. Synthesis of K intercalated coronene

Synthesis at the nominal K_3_coronene composition by reaction of K metal and coronene at 573 K has been claimed to lead to superconducting behaviour with a *T*_C_ ranging from 3.5–15 K, with a shielding fraction of ∼0.5%.^6^ However, no experimental determination of the structure has been reported in any studies under these synthetic conditions. Previous structurally characterized metal-coronene compounds were on the other hand synthesized in the presence of crown ethers, dimethoxyethane (DME), or tetrahydrofuran (THF), wherein the solvents coordinate the potassium cations and enable lower temperature synthesis, but are present in the final product, entirely changing the accessible structural space.^23–26^ Solvent-free synthesis of metal intercalated coronene with detailed structural description of any crystalline phases present is not reported.

Guided by the DFT results, synthesis at the nominal ratio of 3 : 1 KH : coronene was investigated initially. Variable temperature powder X-ray diffraction (VT-PXRD) data were measured *in situ* on a 3 : 1 KH : coronene mixture at 303 K, 423 K, 473 K and 523 K, held for 10 minutes with a measurement period of 1 hour at each temperature (Fig. S4, ESI†). This reveals the formation of a new crystalline phase (reflections marked by *) at 473 K. As the temperature is increased to 523 K, the peaks of the new phase become dominant, with KH Bragg reflections having low intensity, and no coronene reflections observed, indicating near complete reaction at this temperature.

The stoichiometry of K_*x*_coronene was further explored by synthesising samples with nominal *x* = 2, 2.5, 3, 4, and 5 at 523 K in an evacuated quartz ampoule. The powder pattern of the reaction run at a 3 : 1 ratio of KH : coronene contains the new crystalline peaks which were observed by VT-XRD (denoted as K_3_coronene, marked as *), with no observable peaks that can be attributed to the starting materials (Fig. S5, ESI†). As the ratio of KH in the starting mixture decreases (*i.e.*, 2.5 : 1 and 2 : 1 ratio of KH : coronene), a second set of new peaks separate from those associated with K_3_coronene are observed, indicating that a mixture of two different phases form for *x* < 3. The intensities of the peaks associated with the secondary phase (denoted K_*x*_coronene′) increase with decreasing KH : coronene ratio (Fig. S5, ESI†). Ratios greater than 3 : 1 (*i.e.*, 4 : 1 and 5 : 1 ratio of KH : coronene) result in unreacted KH in the product (Fig. S5, ESI†) alongside the peaks associated with K_3_coronene. Therefore, the nominal ratio of 3 : 1 (KH : coronene) and reaction temperature of 523 K for two durations of 6 hours with intermediate regrinding, are the optimal synthesis conditions for the formation of K_3_coronene. Reactions using K metal and coronene at the same compositions yielded amorphized products along with KH (Fig. S6 and S7, ESI†), highlighting the limitation of using strong reducing agents in the synthesis of intercalated PAHs, as this can lead to cleavage of the C–H *σ* bond and the formation of KH. Therefore, no further characterizations and measurements were done for K-metal:coronene products.

**Fig. 3:**
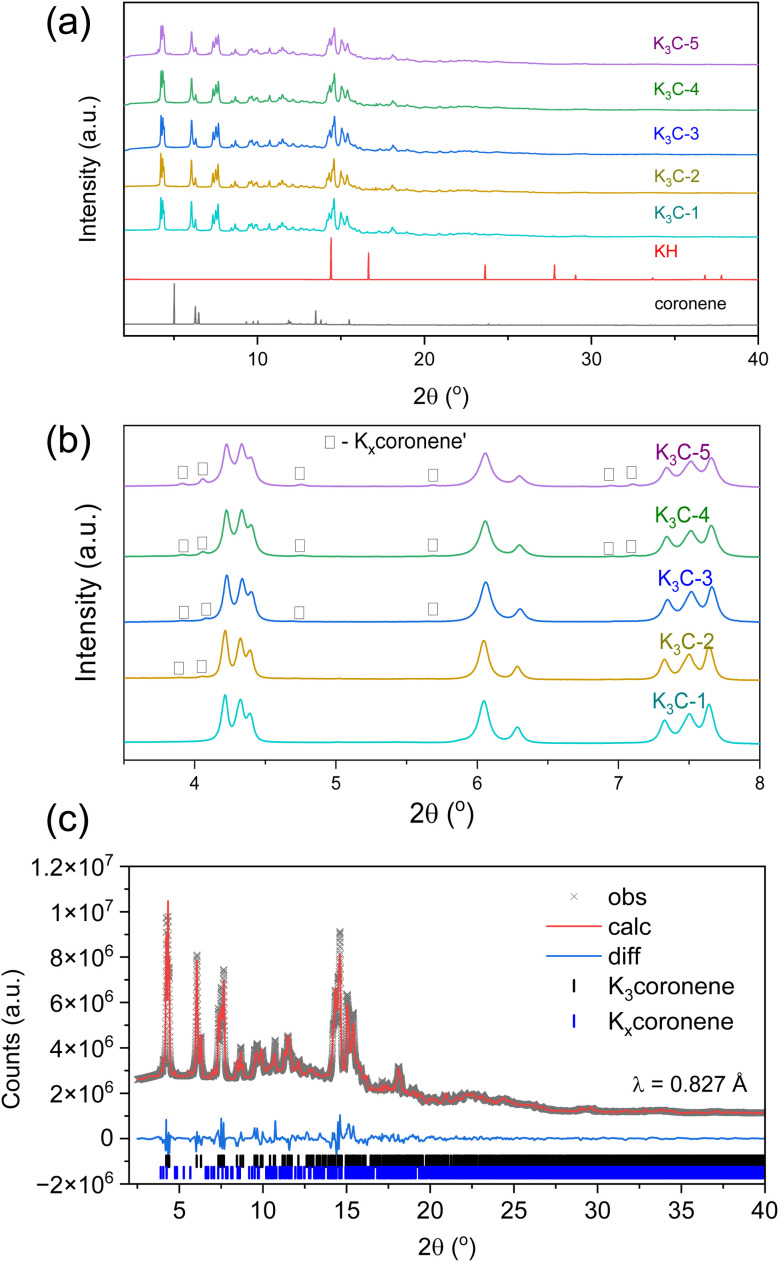
(a) Room temperature PXRD of different batches of K_3_coronene, K_3_cor-1 to K_3_cor-5. No peaks of unreacted coronene or KH are observed. (b) Enlarged PXRD between 3.5°–8.0°. Major peaks correspond to main crystalline phase K_3_coronene, with lower intensity peaks indicating a minor second phase K_*x*_coronene′ (marked by □) with K_3_cor-1 having no independent reflections corresponding to the K_*x*_coronene′ impurity (note, that is it is possible that a small amount of K_*x*_coronene′ remains in this sample). PXRD data were collected on beamline I11 at Diamond Light source, UK (*λ* ∼0.825 Å). (c) Rietveld refinement of K_3_coronene (K_3_cor-4). The phase K_*x*_coronene′ is Pawley fitted to account for the secondary peaks whose highest peak reflection is around 3.4(8) % of the highest peak reflection of the main phase. The *R*_wp_ of the fit is 3.8% for 111 refined parameters.

Fig. 3 shows the PXRD patterns of five different batches of K_3_coronene (labelled as K_3_cor-1 to K_3_cor-5), all of which were prepared using the same synthetic protocol of solid state grinding and annealing for 6 hours at 523 K (see Methods in ESI†). All the batches show a majority crystalline phase of K_3_coronene with no unreacted KH or coronene and, except K_3_cor-1, a small amount of K_*x*_coronene′ (marked by □). The strongest Bragg reflection from K_*x*_coronene′ is 1.4(5) %, 2.2(4) %, 3.4(8) %, 6.1(7) % of the strongest reflection from K_3_coronene for K_3_cor-2 to K_3_cor-5 respectively. In addition to K_3_coronene and K_*x*_coronene′, each sample contains varied amounts of an amorphous phase (quantified using internal diamond standard^27^) ranging from 0.0 wt% for K_3_cor-2 to 27.4 wt% for K_3_cor-4 (Table S2, Section S4, ESI†). This illustrates the challenge in isolating a phase pure powder of K_3_coronene due to competing side reactions to produce secondary crystalline phase(s) or amorphous phases, and is highly dependent on the characteristics of individual reactions, possibly due to a combination of variances in hand-ground mixing and weighing discrepancies (±0.2 mg) between samples (Table S2†). From these synthesis outcomes, it can be concluded that a 3 : 1 ratio of KH and coronene gives a single crystalline phase that can be isolated nearly free from amorphous and other crystalline components but is prone to undergo decomposition at temperatures above 473 K (Fig. S6, ESI†), suggesting metastability and sensitivity to precise reaction conditions that is challenging to fully control.

Elemental analysis (using ICP and CHN) of K_3_cor-1 to K_3_cor-5 is close to the nominal composition (Table S2, Section S4, ESI†). To understand the fate of the coronene molecule during the synthesis process, mass spectroscopy (MS) and ^1^H-Nuclear Magnetic Resonance (^1^H-NMR) spectroscopy were performed on K_3_coronene samples that had been oxidised by controlled exposure to air (see Methods in ESI†). From MS we observe the most intense peak at 301.10 *m*/*z* corresponding to C_24_H_13_^+^ (coronene, C_24_H_12_, molecular weight: 300.1 g mol^−1^) (Fig. S8, Section S5, ESI†). ^1^H-NMR of oxidised K_3_coronene, using toluene-d_8_ as solvent, shows a singlet at chemical shift (*δ*) = 8.65 ppm as the most intense peak belonging to hydrogens in the coronene molecule (coronene has only 1 type of H present, manifested as a singlet) alongside minor peaks due to the side hydrogenation reaction of coronene to form 1,2 dihydrocoronene (Fig. S10, Section S5, ESI†). From both MS and ^1^H-NMR, we see that the majority of the coronene molecules remain intact during the synthetic process, with a minor hydrogenation by-product. Given the low solubility of coronene in toluene-d_8_, the exact amount of 1,2 dihydrocoronene cannot be ascertained. However, the total amount of the hydrogenation by-product is small (<5 mol%) given the low intensity of the extra peaks in the ^1^H-NMR spectrum (Table S3, ESI†) and absence from the MS (see Section S5 in ESI† for more details regarding MS and ^1^H-NMR).

#### 2.2.1. Crystal structure of K_3_coronene

The crystal structure of K_3_coronene was solved using the synchrotron PXRD data collected on sample K_3_cor-4 (see ESI†). The PXRD pattern of the primary K_3_coronene phase was indexed in the monoclinic lattice with space group *C*2 and cell parameters, *a* = 15.0524(12) Å, *b* = 21.892(2) Å, *c* = 13.3640(14) Å and *β* = 122.917(5)° (Table 1 and ESI†) using Topas academic^28^ (Fig. 3c). K_*x*_coronene′ was indexed in space group *C*c with cell parameters, *a* = 41.789(9) Å, *b* = 13.227(4) Å, *c* = 20.853(3) Å and *β* = 34.065(5)°. Structure solution of the main K_3_coronene phase was achieved by simulated annealing of an initial model containing four rigid coronene molecules and 15 K sites (ESI†) and subsequent Rietveld refinement, which resulted in four K sites and one coronene site being removed from the model. The structure solution and Rietveld refinement was obtained using synchrotron PXRD data over the *d*-spacing range 18.95–1.20 Å.

**Table 1:** Crystallographic data for parent coronene, K_3.03(3)_coronene and K_*x*_coronene′

Compound	Coronene	K_3.03(3)_coronene	K_*x*_coronene′
Space group	*P*2_1_/*a*	*C*2	*Cc*
*a* (Å)	16.119(6)	15.0524(12)	41.789(9)
*b* (Å)	4.702(4)	21.892(2)	13.227(4)
*c* (Å)	10.102(6)	13.3640(14)	20.853(3)
*β* (^o^)	110.9(1)	122.917(5)	34.065(5)
Vol (Å^3^)	715.2(9)	3696.1(6)	6457(3)
Source	Ref. 21	Rietveld	Pawley

**Fig. 4:**
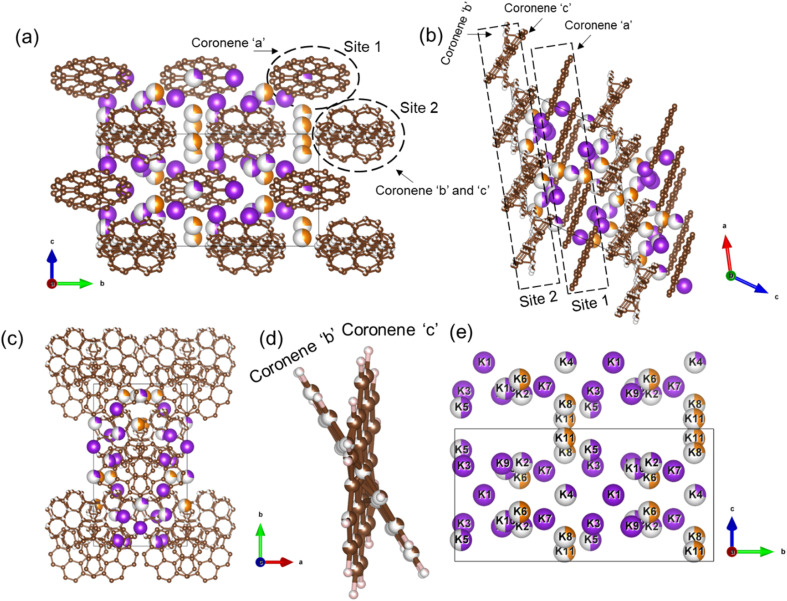
(a), (b) and (c) Structure of K_3.03(3)_coronene viewed along *a*, *b* and *c*-directions, respectively. The structure consists of ordered coronene on site 1 (coronene ‘a’) and disordered coronenes (coronene ‘b’ and coronene ‘c’) on site 2 stacked along the *a*-axis. Brown and pink spheres represent carbon and hydrogen, respectively. The purple and orange spheres represent K^+^ ions present within each chain and in between chains, respectively, with the occupancies represented by the proportion of coloured area on each site. (d) Enlarged view of the disordered site 2 coronenes with occupancies of 0.25(10) and 0.75(10) for coronene ‘b’ and coronene ‘c’. (e) K^+^ positions in the lattice viewed along the crystallographic *a*-direction. Partial occupancy is indicated by the fill fraction of each sphere.

The final refined structure contains two independent coronene sites, both on general positions. One site (denoted site 1) contains a single fully occupied ordered molecule (coronene ‘a’), while the second site (site 2) accommodates two disordered molecules (coronene ‘b’ and ‘c’, with refined occupancies of 0.25 (10) and 0.75 (10) respectively). The disordered coronene molecules are centred on the same co-ordinates but rotate independently of each other (Fig. 4a–d). The K sites are generally disordered within the structure and were all located using simulated annealing during the structure solution (see ESI†). The structure has 11 occupied K sites, nine of which are on the general 4c position, with the remaining two on the 2b position, four of which are fully occupied (K1, K3, K7 and K9), three with a refined occupancy of 0.5 (K5, K6 and K10), one with a refined occupancy of 0.41 (K11), with the remaining three sites having refined occupancies between 0.25–0.28 (K8, K4 and K2) (Fig. 4e). The refined structure differs significantly from the computed structure of K_3_coronene, displaying even greater disruption of the original herringbone coronene packing driven by intercalation of the K^+^ ions. The overall refined composition is found to be K_3.03(3)_coronene which is close to the composition obtained from ICP and CHN (Table S2, ESI†).

**Fig. 5:**
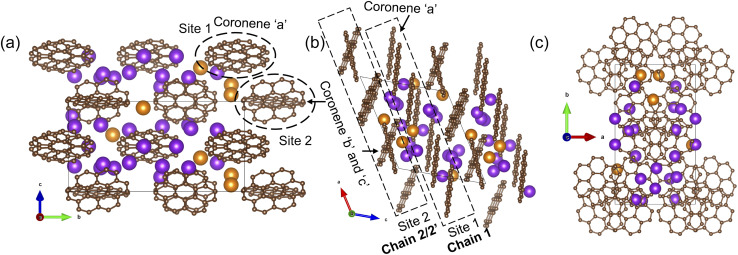
(a)–(c) A locally represented model of the structure of K_3.03(3)_coronene, derived from the disordered refined K_3.03(3)_coronene model. The rounding of K occupancies when assembling the local representation gives the composition K_3.125_coronene. Brown and pink spheres represent carbon and hydrogen, respectively. Purple and orange spheres represent K^+^ ions present within a chain and between the chains, respectively (see Fig. 6).

Given the disordered coronenes (coronene ‘b’ and coronene ‘c’, Fig. 4d) are in the ratio of 0.25 (10) : 0.75 (10), a locally represented model can be assembled where they are periodically arranged in a 1 : 3 ratio, within one unit cell. As each of the two coronene sites are on the general 4c position, this generates a total of 8 coronene molecules in the full unit cell. For site 1, the coronenes (coronene ‘a’) are fully ordered and thus contribute the first four molecules to the model. For site 2, the first molecule was set in the ‘*b*’ orientation, with the remaining three in the ‘*c*’ orientation, maintaining the refined ratio (Fig. 5a–c). When building this model, for each K site that does not have an occupancy of either 1, 0.5 or 0.25, their occupancies are rounded to whichever of these values is nearest (for example, K11, which has an occupancy of 0.41, is rounded to 0.5), resulting in a model with a composition of K_3.125_coronene. For the K2 and K8 sites, of the four sites generated, only the sites around the ‘a’ coronene generated from site 1 were retained, maintaining their rounded occupancy of 0.25. For the remaining partially occupied K^+^ sites, the corresponding number of K^+^ sites were retained to maintain the stoichiometry of the structure (*e.g.*, half of the sites were retained for occupancy of 0.5, and a quarter of the sites generated for the remaining site with an occupancy of 0.25). The choice of which sites to retain was made retaining physically reasonable distances between neighbouring K^+^ sites and the individual coronene molecules. The distances and angles between the K^+^ ions and the centre of the closest aromatic ring of coronene molecules in the locally represented model are given in Fig. S11, ESI and Table S4, ESI.† Each K^+^ ion is bound to multiple coronene molecules, although the co-ordination is off-centre. Each K^+^ is closer to one coronene molecule, except for K2, which is equidistant to two neighbouring coronene molecules, the bonding to the closest coronene is shown for each K^+^ in Fig. S12.† The off centre co-ordination of the K^+^ ions is similar to that observed in K_2_picene. These distances and angles are comparable with the other K–PAH structures that have been reported, indicating the interactions in K_3_coronene are similar to the other K intercalated PAHs studied.^29^

**Fig. 6:**
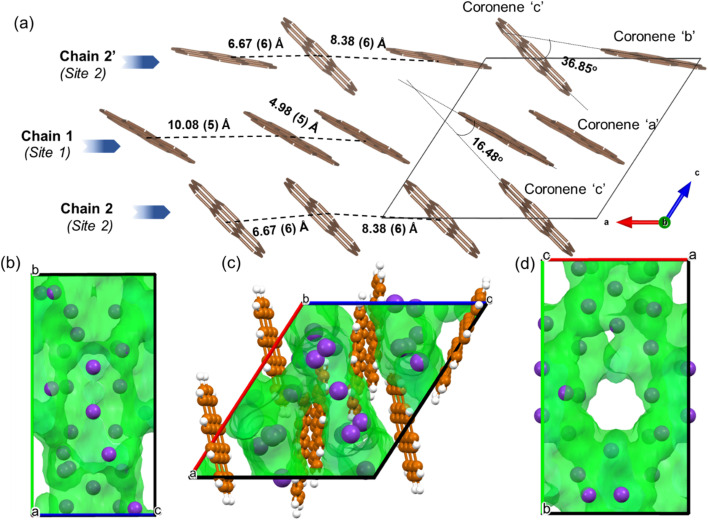
(a) Locally represented model of K_3_coronene derived from the refined disordered structure shown in Fig. 4, showing the different chains formed along the *a*-axis. The distances between the centres of coronene molecules along the chain and the angles between different coronene molecules are also shown. The K^+^ ions intercalate both within and between the chains, and are shown in Fig. S14–S17, ESI.† (b–d) Void space of the locally represented model of K_3_coronene computed and plotted using Mercury^20^ with a probe radius of 1.0 Å along *a*-, *b*- and *c*-crystallographic directions respectively. Coronene molecules are removed in (b) and (d) to get a clearer view of the void space. The K atoms (purple spheres) reside inside the computed voids (green area).

In the locally represented model, coronene stacks in three types of chains along the *a*-axis. Chain 1 consists of ordered coronene in site 1 (coronene ‘a’) with alternating centre-to-centre spacing of 4.98 (5) Å and 10.08 (5) Å (Fig. 6a) (here “centre” refers to the geometric centre of each coronene molecule), compared with 4.70 Å in pristine coronene. This alternation arises from the insertion of K^+^ ions (K1, K3, K4, K7) between every pair of coronene molecules in the chain creating the 10.08(5) Å separation, with a 4.98(5) Å separation within the pairs themselves (Fig. S14, Section S6, ESI†). Chain 1 retains the same face-to-face orientation of coronene molecules observed in pristine coronene, but with the alternating spacing between pairs of molecules to allow for the intercalation of K^+^ ions. In chain 2, coronene ‘c’ from site 2 stacks along *a*-axis with alternating 8.38(6) Å and 6.67(6) Å spacings. Similarly, chain 2′ comprises alternating coronene ‘b’ and coronene ‘c’ molecules, with alternating 8.38(6) Å and 6.67(6) Å separation (Fig. 6a). In both chains 2 and 2′, there are intercalated K^+^ between every coronene molecule (Fig. S15 and S16, ESI†), in contrast to the presence of coronene pairs in chain 1. Apart from these K^+^ ions located within the chains, the remaining K^+^ ions (K6, K8 and K11) reside between the chains as shown in Fig. S17, ESI.† The orientation of molecules in both variations of chain 2 do not follow that observed in pristine coronene.

In crystalline K_2_pentacene, K_2_picene K_2_rubrene and K_2_tetracene, the predominant C–H⋯π interactions that were present in the structure of the parent molecule were found intact after incorporation of the K^+^ ions, with formation of a large void within the herringbone chains, *via* simple rotation around the molecular axis (Fig. S18 and S19, ESI†).^9,11,12^ These voids can only accommodate ∼2 K^+^ ions per PAH molecule. Attempts to synthesise phases with a higher ratio (>2 : 1 of KH : PAH) of K^+^ ions resulted in unreacted KH in the product mixture for picene and pentacene,^9^ while for K^+^-intercalated rubrene, they resulted in a significant amount of a secondary phase K_*x*_R′.^11^ For K^+^ ion intercalated tetracene, there are only two voids that K^+^ ions can occupy and therefore, cannot physically intercalate a higher ratio (>2) of K^+^ ions.^12^ In the parent crystalline coronene, the coronene molecules are also packed in a herringbone fashion similar to picene and pentacene (Fig. 2b).^21^ However, unlike any previously reported K^+^ intercalated PAHs, the formation of a K^+^ ion intercalated phase in coronene is possible at a ratio of ∼3 : 1 between KH and coronene. Given pristine coronene has similar porosity, calculated by Zeo++, to the other PAHs mentioned (Table S1, ESI†), intercalation of more K^+^ ions per molecule than the other PAHs required a more extensive reorientation of the coronene molecules in order to create multiple pockets within the chains, thereby disrupting its original herringbone motif. In pristine coronene, the angle between the two coronene molecules in neighbouring chains is 85.42° (Fig. 2b), whereas after the K^+^ ions intercalation and subsequent reorientation of the coronene molecules, the angles between the coronene molecules decreases to 36.85° between coronene molecules ‘b’ and ‘c’ and 16.48° between coronene molecules ‘a’ and ‘c’ (Fig. 6a). In contrast the angles between molecules in pristine picene (57.89°), pentacene (52.9°), and tetracene (51.4°) can rotate to open up more void space in K_2_pentacene, K_2_picene K_2_rubrene and K_2_tetracene where the molecules in alternating chains align at ∼90° to each other.^9,11,12^ K^+^ intercalation in coronene results in the extensive reorientation of chain 2/2′ giving coronene molecules with much shallower angles between chain 1 and chain 2/2′ (Fig. 4a–c). The reorientation of coronene molecules increases the void fraction (the percentage of the unit cell calculated to be void space) from 4.3% of the unit cell volume in the parent to 22.7% allowing the incorporation of three K^+^ ions per coronene molecule. The void fraction in K_3.03(3)_coronene is higher compared to K_2_picene (6.0% of the unit cell) and K_2_pentacene (12.5% of the unit cell), thereby affording the larger number of K^+^ ions within the cell, reflecting the much greater disruption to the original herringbone packing in the original coronene case. When incorporating 3 K^+^ ions into C_60_ its void fraction increases from 13% in the pristine material to 19.4% in K_3_C_60_, a much smaller increase overall compared to K_3.03(3)_coronene but with a similar value for the intercalated materials, indicating the larger void fraction necessary to accommodate 3 K^+^ ions and manifesting *via* the disruption to the original herringbone packing.

**Fig. 7:**
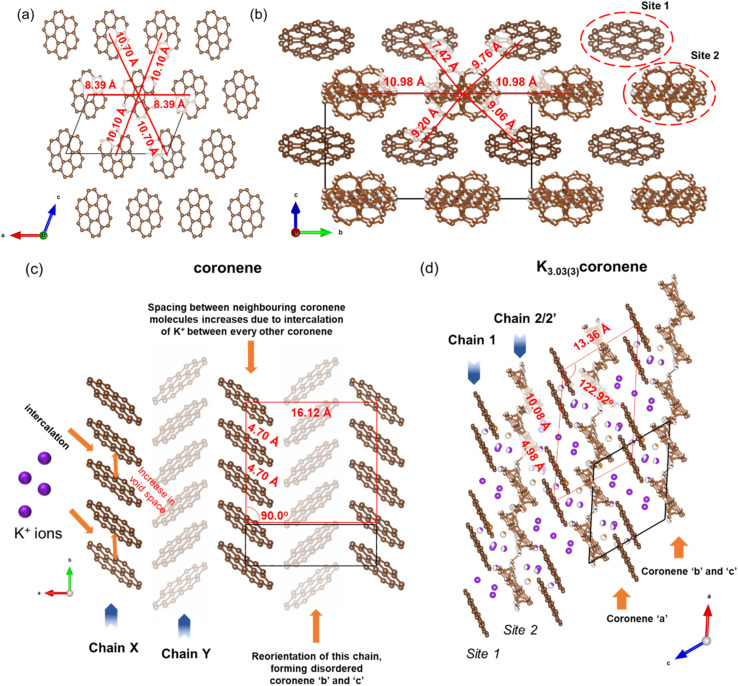
(a) Packing of different chains in pristine coronene along the *b*-axis. The chains consist of equivalent coronene molecules. Each coronene chain is surrounded by six neighbouring chains as depicted by their respective distances. The brown spheres represent the carbon atoms of coronene. Hydrogens are removed for clarity. (b) Packing of different chains in K_3.03(3)_coronene viewed along the *a*-axis (equivalent to the *b*-axis in coronene). Similar to coronene, each chain in K_3.03(3)_coronene is also surrounded by six neighbouring chains. The K^+^ ions are removed for clarity. Chains including K^+^ ions positions are shown in Fig. S20, ESI.† (c) Crystal structure of coronene viewed along the *c*-axis, and (d) crystal structure of K_3.03(3)_coronene viewed along the *b*-axis. Chain 1 consists of a larger coronene–coronene centre-to-centre distance between a molecular pair separated by K^+^ ions that alternates with a shorter distance which does not contain such K^+^ ions. The motif of coronene orientations in chain 1 resembles that of chain X in pristine coronene while chain 2/2′ reorients and forms a disordered chain in order to accommodate the K^+^ ions (purple spheres represent K^+^ ions within the chain and orange spheres between the chains). While disordered in the experimental structure, chain 2/2′ form two distinct chain types when a locally represented model was determined. The red boxes in (c) and (d) are representative of the packing of coronene with the vertices placed at either end of a chain of four coronene molecules in Chain X (coronene) and Chain 1 (K_3.03(3)_coronene), which is heavily distorted upon K^+^ intercalation, with alternating short and long coronene–coronene distances (the box does not represent the unit cell, the unit cell is given by the black solid lines).

After incorporation of K^+^ ions in coronene, although there is disruption to the herringbone packing, part of the original motif remains intact. The chains in pristine coronene are stacked along the crystallographic *b*-axis, with each chain being surround by six neighbouring chains in the *ac* plane (Fig. 7a). In K_3.03(3)_coronene, although we see reorientation of the coronene molecules, the close packing of the chains is retained, with each chain being similarly surrounded by six neighbouring chains (Fig. 7b). The retention of packing motif between chains upon K^+^ intercalation is also observed for picene and pentacene (Fig. 7a, b and S19, ESI†).

In chain 1 in K_3.03(3)_coronene, consisting only of ordered coronene ‘a’, the molecular arrangement resembles that of chain X in pristine coronene (Fig. 7c, d and S20, ESI†). The larger coronene–coronene centre-to-centre distance of 10.08(5) Å is due to the intercalation of K^+^ ions between pairs of coronene molecules. The alternate shorter coronene–coronene centre distance of 4.98(5) Å in chain 1, between which there are no intercalating K^+^ ions, is close to the distance of 4.70 Å in pristine coronene. Despite their alternating long and short distances, their orientation remains similar to that of pristine coronene (Fig. 7c, d and S20, ESI†). The other chain of pristine coronene (chain Y), however, undergoes a reorientation and becomes disordered forming chain 2/2′ in K_3.03(3)_coronene, together with an increase in the molecular separation to accommodate the K^+^ ions between every coronene in the chain.

#### 2.2.2. ^13^C cross polarization magic angle spinning nuclear magnetic resonance (CP MAS NMR)

**Fig. 8:**
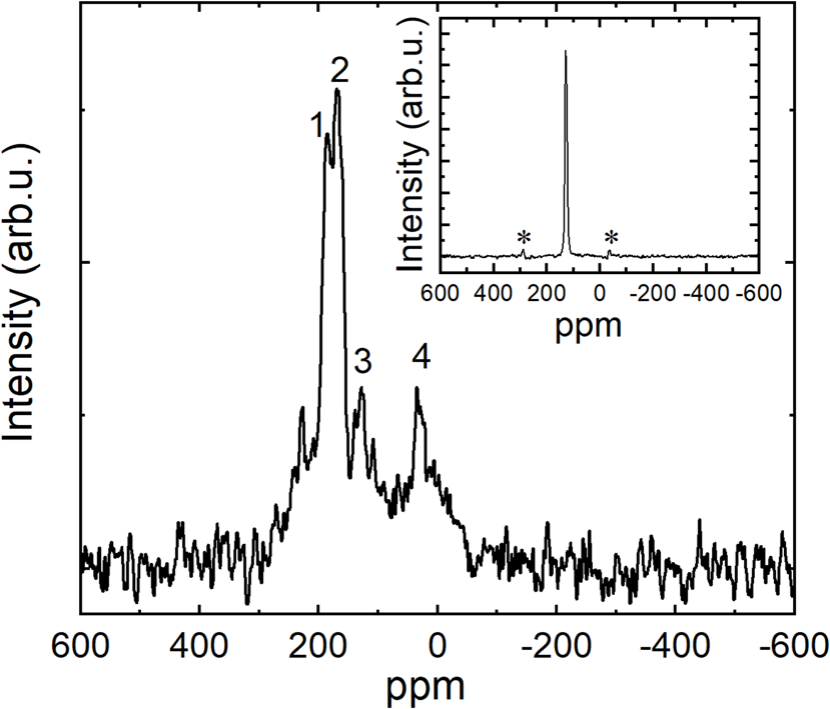
^13^C CP MAS NMR spectrum of K_3_coronene powder (K_3_cor-1) measured at room temperature. Numbers from 1 to 4 label peaks with shifts of 186.14 ppm, 167.05 ppm, 128.13 ppm and 30.76 ppm relative to TMS standard, respectively. (inset) ^13^C CP MAS NMR spectrum of parent pure coronene powder. The measured shift is 128.14 ppm. The sample was rotated at 20 kHz, the observed sidebands marked with ‘*’ in inset are MAS NMR experimental artefacts.

The ^13^C Cross Polarization Magic Angle Spinning Nuclear Magnetic Resonance (CP MAS NMR) of K_3_coronene (K_3_cor-1) powder shows 4 distinct peaks (Fig. 8). A doublet of peaks, labelled as peaks 1 and 2 (27% of total signal intensity), with shifts of 186.14 ppm and 167.05 ppm are far off from the chemical shift of *σ*_CS_ = 128.14 ppm of pure coronene (Fig. 8 (inset)). The additional shifts of peaks 1 and 2 by 58 ppm and 38.91 ppm relative to pure coronene, are in the same range as those observed in alkali-doped fullerides^30,31^ and are thus attributed to the contact hyperfine coupling to the unpaired spin density on these coronene molecules. This at the same time explains their very short spin-lattice relaxation times, *T*_1_, of 58 ms and 77 ms, respectively. These peaks are thus assigned to the inequivalent coronene molecules on site 1 and site 2. On the other hand, peak 3 (4% of total signal intensity) has a shift of 128.13 ppm and is thus centred at *σ*_CS_ for pure coronene. This implies that coronene molecules without additional contact hyperfine shift are present in the NMR sample. To check on whether their presence is intrinsic or due to the sample aging effects, we measured ^13^C MAS NMR spectrum of the same sample after 14 days. In the aged sample we find a significant increase of peak 3 and simultaneous decrease in peaks 1 and 2 (Fig. S21, ESI†) suggesting that the presence of coronene peak 3 is due to the slow sample degradation by atmospheric oxidation in the incompletely sealed NMR rotor. The spin-lattice relaxation time of peak 3, *T*_1_ = 200 ms, is significantly longer than those of peaks 1 and 2, however still an order of magnitude shorter than what we measured for the pure coronene sample (*T*_1_ = 1.8 s). The faster relaxation of ^13^C spins of coronene molecules in our sample could be due to neighbouring unpaired spins of the K_3_coronene. Finally, peak 4 is measured at the shift of 30.76 ppm and has the longest *T*_1_ of ∼1 s. It is thus attributed to the amorphous phase present in several K_3_coronene samples. Besides these four distinctive peaks, there is sizeable NMR signal intensity in the broad background underlying the four peaks, which could be attributed to the presence of structural disorder in our sample (Fig. 4a–c). Additionally, there is also a weak peak at 228.44 ppm with *T*_1_ = 89 ms, which represents only about 1% of the total signal intensity. The origin of this small signal is currently unknown.

### 2.3 Magnetic measurements

K_*x*_coronene (*x* = 2.5–3.5) phases are reported to be superconducting below 15 K.^6^ To probe the possible superconductivity we measured the Zero Field Cooled (ZFC) and Field Cooled (FC) magnetization at an applied field of 10 mT using a superconducting quantum interference device (SQUID). In disagreement with the literature data,^5^ the ZFC-FC data do not show any evidence of superconductivity across all the different batches of K_3_coronene in the measured temperature range of 2–300 K (Fig. S22, ESI†).

**Fig. 9:**
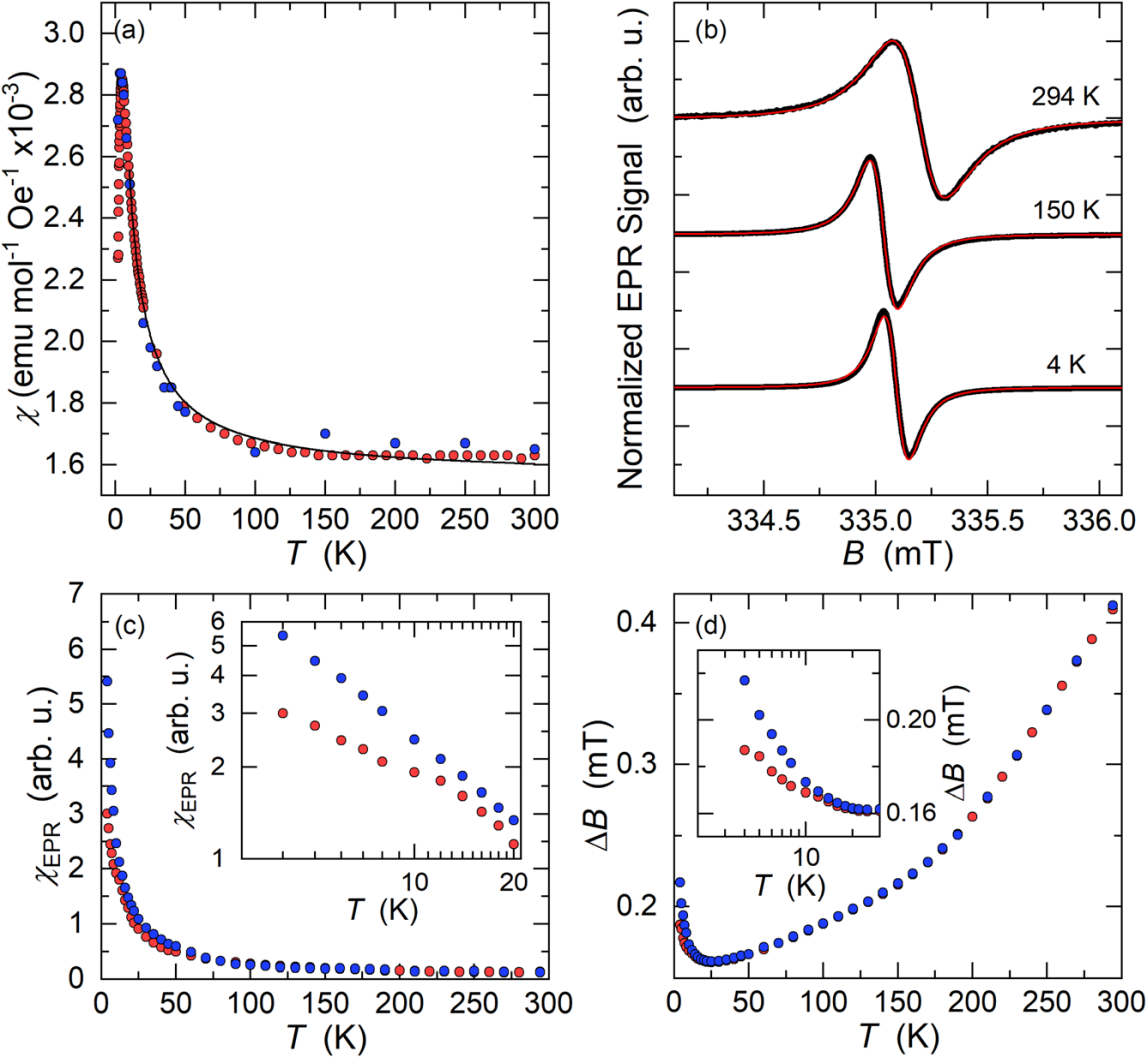
(a) Temperature dependent magnetic susceptibility (*χ*) of K_3_coronene (K_3_cor-1) measured with a SQUID magnetometer (2–300 K). *χ*(*T*) values denoted by red circles are obtained from the magnetic moment difference measured at applied fields of 6.5 T and 5.5 T and corrected for core diamagnetism. Blue circles are the susceptibility values obtained from the high-field (5–7 T) slope of M(H) isotherms. The *χ* is fitted with modified Curie–Weiss law in the temperature range of 10–300 K (black line). (b) X-band EPR spectra of K_3_cor-1 powder (black lines) measured at three characteristic temperatures. The solid red line is a spectral fit to a single Lorentzian lineshape. (c) Temperature dependence of the EPR spin susceptibility, *χ*_EPR_, measured on slow cooling (blue circles) and on warming after rapidly cooling the sample to *T* = 4 K (red circles). The inset shows the expanded low-temperature region in the logarithmic scale. (d) The temperature dependence of EPR linewidth, Δ*B*, measured on slow cooling (blue circles) and on warming after rapid cooling (red circles). The inset shows the expanded low-temperature region in the logarithmic scale.

The as-measured ZFC-FC susceptibility data show a divergence in their temperature dependence below 300 K indicative of ferromagnetic impurities. The starting material coronene, when measured without any previous thermal treatment, shows diamagnetism. A similar ZFC-FC divergence to K_3_coronene (Fig. S23a, ESI†) is observed for pristine coronene after treatment with the temperature profile used in the synthesis of K_3_coronene (Fig. S23b, ESI†). To remove the contributions from these unknown ferromagnetic impurities, the 2–300 K magnetic susceptibility (*χ*) was determined as the difference in the high field magnetic moment at applied fields of 6.5 and 5.5 T (Fig. 9a). The magnetic susceptibility obtained in this way is comparable with the susceptibility values obtained directly from the M(H) isotherms. Field-dependent magnetization isotherms were also measured on samples K_3_cor-1 between 2–300 K from 0 to 7 T (Fig. S24†). The susceptibility was obtained from the slope of the linear component between 5–7 T (see methods in ESI†). The susceptibility increases with decreasing temperature, has a maximum at 4.5 K and then decreases with temperature upon cooling. We note though that the maximum in *χ* may be artificial as it could result from paramagnetic saturation at high fields and low temperatures affecting the extracted magnetic susceptibility.

The susceptibility data fitted with a modified form of the Curie–Weiss law^32^ taking into account the temperature-independent background contributions (*χ*_0_), in the temperature range of 10–300 K, yields a Curie–Weiss temperature (*θ*_CW_) of −3.0 (3) K indicative of antiferromagnetic interactions. The Curie constant, *C* is found to be 0.0128 (4) emu K mol^−1^ Oe^−1^ corresponding to an effective magnetic moment, *μ*_eff_ of 0.319 (4) *μ*_B_ per f.u. The number of unpaired electrons (*n*) is then calculated using the equation, 
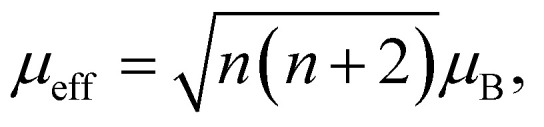
 and corresponds to 0.05 unpaired electrons per coronene molecule. The temperature independent term *χ*_0_ is found to be 15.60 (4) × 10^−4^ emu mol^−1^ Oe^−1^. Should *χ*_0_ indicate Pauli susceptibility of the metallic K_3_coronene phase, then this phase would be characterised by a large density of states at the Fermi energy that is comparable with Rb_2_CsC_60_ (*χ*_0_ = 7 × 10^−4^ emu mol^−1^)^31^ and K_3_C_60_ (*χ*_0_ = 3.8 × 10^−4^ emu mol^−1^).^33^ As this is very unlikely even for a narrow band correlated system and is not corroborated by EPR spectra (*vide infra*) we conclude that large *χ*_0_ probably results from our difficulties in complete subtraction of the ferromagnetic impurity contributions. All the fitting parameters are given in Table S5, Section S7, ESI.†

Electron paramagnetic resonance (EPR) spectroscopy, which is sensitive to the magnetic coronene molecules only, is performed for a more detailed magnetic characterization. At room temperature, we observe an intense X-band EPR signal for all K_3_coronene samples (Fig. 9b and S25, Section S7, ESI†). The EPR spectrum is centred at *g* = 2.0033, as expected for a PAH molecular system with a very weak spin–orbit coupling. A very small deviation of measured *g*-factor from the free-electron value *g*_e_ = 2.0023 suggests that the EPR response is dictated largely by the coronene molecular orbitals and that the remaining s-electron density on the potassium metals must be very small.^34^ The measured EPR signal does not show any significant lineshape anisotropy that would be attributed to the Dyson lineshape typically found in metallic samples. ^35^ A fit to a Lorentzian lineshape (Fig. 9b) yields the room-temperature EPR linewidth of Δ*B* = 0.40 ± 0.01 mT while the EPR signal intensity calibration shows that only around 10% of the coronene molecules contribute to the measured signal which is close to the 5% of unpaired spins estimated from SQUID magnetometry.

On cooling, the intensity of EPR signal, *χ*_EPR_, increases with decreasing temperature, initially approximately following the Curie–Weiss law between room temperature and ∼20 K (Fig. 9c). This agrees with the insulating nature of the K_3_coronene compound and corroborates the absence of Dyson-like lineshape asymmetry (Fig. 9b). The EPR linewidth first decreases with decreasing temperature down to 25 K where Δ*B* reduces to 0.16 ± 0.01 mT (Fig. 9d). We notice that in the large temperature interval between 50 K and 150 K, the EPR linewidth follows linear temperature dependence. In the same temperature interval between room temperature and 25 K, the *g*-factor remains constant within the experimental precision. However, below ∼20 K, we start to observe deviations from the high-temperature trends in all EPR parameters. First, *χ*_EPR_ becomes dependent on the precise cooling protocol that is used. For example, in measurements taken after rapidly cooling the sample to the lowest temperature of *T* = 4 K, *χ*_EPR_ is suppressed compared to the Curie–Weiss-like dependence, but above ∼20 K it coincides with the data taken in the slow cooling experiments. Similarly, the EPR spectra start to broaden on cooling below ∼25 K and Δ*B* increases to 0.22 ± 0.01 mT at 4 K (Fig. 9d) in the slow cooling experiment. On the other hand, the broadening (Δ*B* = 0.19 ± 0.01 mT at 4 K) is less pronounced if the sample has been initially rapidly cooled.

The almost ideal Lorentzian lineshape of EPR spectra and the Curie–Weiss-like temperature dependence of EPR spin susceptibility confirm the insulating state of K_3_coronene. This speaks for the charge localisation on orientationally disordered coronene molecules. The two main mechanisms for the charge localisation are the disorder-induced localisation in Anderson insulators and electron–electron driven localisation in Mott insulators.^36^ As both the disorder and electron correlations are present in the investigated system, the emerging magnetic state may be especially complex.

Yet the small magnitude of EPR spin susceptibility of K_3_coronene containing formal coronene^3−^ units is surprising from the perspective of molecular orbital energies (Fig. 1b). Our SQUID and EPR data exclude itinerant paramagnetism and the magnetic response is compatible with the local moments. Structural motifs, especially for the chains 1 and 2′, comprise two coronene molecules in close contact (Fig. S13, ESI†). Model DFT calculations of exchange coupling of coronene^3−^ pairs show that the singlet state is their ground state, but the exact details of exchange depend on the relative molecular shift and tilt (see Section S8 DFT calculations in ESI†). Therefore, there is a strong tendency for singlet formation at the observed coronene separations and the structural motifs determined for K_3.03(3)_coronene are consistent with complete moment suppression. This may then explain the extensive magnetic moment loss, which is consistently observed in both SQUID and EPR measurements on multiple samples where the chemical composition, phase purity and crystalline content are consistent. However, as the structure itself is heavily disordered (Fig. 4a–c), the optimal coronene^3−^–coronene^3−^ contact is not satisfied in all cases, *e.g.*, for chain 2′, and thus some of the unpaired coronene^3−^ spins are recovered and detected in our experiments. The studied K_3_coronene structure is too disordered to reliably identify all the specific pairwise interactions that in reality contribute to the balance between singlet coronene dimers and coronene^3−^ free moments. The extensive disorder and the range of relative molecular shifts and tilts that will combat this align with our robust observation of incomplete moment suppression in EPR and SQUID measurements.

## 3. Conclusion

We have provided a systematic methodology to screen PAHs which can potentially intercalate alkali metals ions. This is important because of the experimental challenges that confront defining suitable synthetic conditions to access crystalline intercalation compounds. Coronene was identified as the preferred candidate with a three step computational prioritisation, comprising (1) screening 195 PAHs, and hierarchically clustering them in terms of their HOMO−1 to LUMO+4 molecular orbital energies, (2) evaluating similarity in the parent crystal structure and calculated porosity with the previously reported K^+^ intercalated PAHs, and (3) DFT calculations on computationally generated intercalated structures to identify the most favourable composition between K and the PAH. As predicted from the theoretical calculations, the 3 : 1 ratio of KH and coronene is synthetically found to be the ideal composition for the formation of a dominant crystalline phase of K_3.03(3)_coronene. Crystal structure prediction calculations anticipated significant rearrangement of the coronene molecules with an increase in coronene–coronene distances from 4.70 Å to 7.91 Å, and the angle between two coronene molecules being increased to 101.24° from 85.42° on K^+^ intercalation.

In contrast to the previously characterized K^+^ intercalated PAHs (namely picene, pentacene, rubrene and tetracene), structural investigation of K_3.03(3)_coronene revealed that the herringbone motif is disrupted by the incorporation of K^+^. Pristine picene, pentacene, rubrene and tetracene all have angles between the molecules in the herringbone structure of 50°–60° (Fig. S1†), and on intercalation these angles increase close to 90° (Fig. S18†) to create the voids for K^+^, but the herringbone motif is not significantly disrupted. In pristine coronene the angle between molecules in the herringbone structure is already close to 90° (85.4°, Fig. 2b) meaning the structural response to intercalation is through the disruption of the herringbone motif, although the chains and their packing are retained. The disrupted herringbone structure allows the intercalation of more K^+^ ions per PAH in coronene compared to the PAH hosts studied previously, reflected in the larger void space. K_3.03(3)_coronene consists of chains of ordered coronene in site 1 and disordered coronene in site 2 along the crystallographic *a*-axis. Despite the apparent disruption of the herringbone packing, after the K^+^ intercalation site 1 is identifiable as retaining the motif of pristine coronene. The K^+^ ions in the lattice reside in the voids formed both within and between chains as a result of the rearrangement of the coronene molecules. Contrary to previous reports, none of the K_3_coronene batches synthesized here show superconductivity in the temperature range of 2–300 K: since there are now an uneven number of electrons transferred per PAH, this must focus attention on still-unidentified amorphous decomposition products as candidate superconductors, or other phenomena as potential sources of the reported negative magnetisation than superconductivity. Magnetic measurements on K_3_coronene are instead consistent with local-moment magnetism where extensive loss of magnetic moments is due to the spin-singlet formation on pairs of nearest neighbouring coronene^3−^ anions.

Given the challenges in synthetic isolation of alkali metal PAH materials, the computationally-enabled workflow applied here can enable experimental effort to be targeted on the most tractable candidates, both in terms of PAH host and intercalation composition. This would assist assessment of newly synthesised PAH as candidate intercalation hosts, and also allow evaluation of the use of multiple metals to occupy the potential inter- and intrachain sites within the herringbone packings studied here. Although K_3.03(3)_coronene is definitively not superconducting, the 3− anion charge and degenerate LUMO indicate that it is possible to attain high-level compositional and electronic similarity with the A_3_C_60_ systems.

## Data availability

Underlaying data can be found on the University of Liverpool Data Repository https://doi.org/10.17638/datacat.liverpool.ac.uk/2642.

## Author contributions

MD and ABC contributed equally to the investigation reported in the manuscript. MD and ABC developed the synthetic protocols and performed the synthesis and characterisation and analysed the data. TK collected and analysed EPR and MAS NMR data. CMC solved the crystal structure. TDM supervised the work, advised on synthesis and characterisation and assisted with data analysis. HN collected magnetic properties data and assisted with data analysis. LMD contributed to analysis of PXRD data. AV performed computational screening experiments. LAJ collected and analysed the solution proton NMR data. BPM assisted with crystal structure analysis and visualisation. YT performed DFT calculations. TWS assisted with analysis of PXRD data. JBC assisted with analysis of crystallography. NGB, MSD, MJR conceptualised the project, obtained funding. MD, TK, TDM, CMC, LMD prepared the original draft and performed review and editing. All authors reviewed and edited the manuscript.

## Conflicts of interest

The authors declare no completing financial interest.

## Supplementary Material

SC-OLF-D4SC05128A-s001

SC-OLF-D4SC05128A-s002
