# Supplementary material for: Multiple cation insertion into a polyaromatic hydrocarbon guided by data and computation
Source: Chem Sci. 2024 Dec 9;16(5):2238–50. doi: 10.1039/d4sc05128a (PMC11697372; doi:10.1039/d4sc05128a)
Supplement: SC-016-D4SC05128A-s001 [file SC-016-D4SC05128A-s001.pdf]

Supplementary Information (SI) for Chemical Science.
This journal is © The Royal Society of Chemistry 2024

Supporting Information

Multiple Cation Insertion into a Polyaromatic Hydrocarbon Guided by Data and Computation

Moinak Dutta,[1,†] Angelos B. Canaj,[1,†] Tilen Knaflič,[2,3] Christopher M. Collins,[1] Troy D. Manning,[1] Hongjun Niu,[1] Luke M. Daniels,[1] Aikaterini Vriza,[1] Luke A. Johnson,[1] Bhupendra P. Mali,[1] Yuri Tanuma,[2] T. Wesley Surta,[1] John B. Claridge,[1] Neil G. Berry,[1] Denis Arčon,[2,4] Matthew S. Dyer[1] and Matthew J. Rosseinsky[1,*]

[1] Department of Chemistry, University of Liverpool, Crown Street, L69 7ZD Liverpool, U.K.

[2] Jožef Stefan Institute, Condensed Matter Physics Department, Jamova SI-1000 Ljubljana, Slovenia.

[3] Institute for the Protection of Cultural Heritage of Slovenia, Research Institute, Poljanska cesta 40, SI-1000 Ljubljana, Slovenia.

[4] University of Ljubljana, Faculty of Mathematics and Physics, Jadranska 19, SI-1000, Ljubljana, Slovenia.

**Email: rossein@liverpool.ac.uk*

Contents:

Section S1. Methods .... (S2)

Section S2. Structure and porosity analysis of different PAHs...............(S8)

Section S3. Variable Temperature and variable composition synthesis of K-coronene… (S12)

Section S4. Elemental Analysis, relative impurity phase and amorphous content……(S17)

Section S5. Mass spectroscopy and ^{1}H-NMR .... (S18)

Section S6. Crystal structure of K_3coronene..................(S21)

Section S7. MAS-NMR and magnetization data …………..(S30)

Section S8. DFT calculations .... (S35)

Section S1. Methods

Starting Materials. Coronene (99%, Sigma Aldrich) and KH (30 wt % dispersion in mineral oil, 99.999%, Sigma Aldrich). Coronene was used as obtained and no further purification was done. KH was extracted from oil by washing it with non-polar solvents under Argon atmosphere.

KH extraction. Oil was removed on Schlenk line. KH dispersed in mineral oil was taken in a two-necked flask attached to Schlenk line. The flask was then purged with Argon gas thrice to ensure removal of Oxygen. Anhydrous toluene (used twice, 50 mL), followed by dry pentane (50 mL) was used as washing reagents to remove the oil. The solvents were mixed with the KH by stirring at room temperature for 20 mins (PTFE coated stirrer was used) and then the solvent was removed via cannula. The KH is then dried under vacuum, ~500 mg dried at a time (Figure 3a, main text). It is then re-purged with Argon and then transferred from the Schlenk line to an Argon filled glove-box and used for the synthesis.

Synthesis of K_3coronene (K_3cor-1 to K_3cor-5). 3:1 ratio of KH and coronene (weights taken for different batches are given in Table S2) were taken in a mortar and then ground for approximately 10 mins inside an argon filled glove box, until the mixture looks homogenous. The mixture is then transferred to a 5 mm diameter quartz tube and vacuum sealed under a pressure of ~ 2 x10^{-5} Torr. The sealed tube is then heated to 523 K at 2 K/min, dwelled for 6 h, and cooled to room temperature by turning off the oven. The quartz tube is then opened inside the globe box and the mixture is then reground for another 10 mins and transferred to a 5 mm diameter quartz tube. The tube is then resealed under vacuum (~ 2×10^{-5} Torr). The quartz tube is again heated to 523 K at 2 K/min, dwelled for 6 h and then cooled to room temperature at 10 K/min. The mixture is then again taken out of the tube inside the glove box and re-ground (~ 10 min) for a third time, sealed under vacuum and then further heated to 523 K at 2 K/min and dwelled for 6h. It is then cooled to room temperature by turning off the oven. The final product is then transferred to a glove box and used for further analysis and measurements.

Powder X-ray Diffraction (PXRD). The compounds were loaded into capillaries of 0.7 mm outer diameter and flame-sealed and checked under a microscope to ensure adequate sealing. PXRD data were collected on I11 beamline (λ ~ 0.825 Å) in Diamond Light Source, UK and using a Cu Bruker (λ ~1.5406 Å). Variable temperature PXRD measurements 303 K, 423 K,

473 K and 523 K are done by mixing appropriate ratio of K-metal/KH and coronene in a glove box, loaded into capillaries of 1.5 mm inner diameter, flame sealed and measured on a Rigaku diffractometer having Mo source ($\lambda \sim 0.71$ Å) with an Anton Parr HTK 1200N high temperature stage. Samples are heated at 2 K/min and held at the target temperature for 10 minutes before beginning the measurement scan (1 hour).

Relative weight% of amorphous phase in K_3coronene. The relative weight% of the amorphous phase in different batches K_3coronene (K_3cor-1 to K_3cor-5) is measured by mixing a known ratio of crystalline diamond (as an internal standard) and K_3coronene and then performing a Rietveld refinement of the mixture.[1] The difference between the wt% of known K_3coronene amount and obtained K_3coronene amount from Rietveld refinement is then accounted for the amorphous phase. We note that as all the samples are likely to contain some component of $K_x$coronene′, the refined amorphous content is likely to be an over estimate, since we do not have a structural model for the phase, it is not included in the weight fraction calculations.

^{1}H - Nuclear Magnetic Resonance (^{1}H NMR) spectroscopy. ^{1}H-NMR was performed by controlled oxidation of the compounds in air, by taking the few mg of the sample out from the glove box and keeping in a fume hood overnight. The oxidized samples were then dissolved in Toluene-d^8. Both coronene and K_3coronene were found to be slightly soluble in Toluene-d^8. The dissolved part was then transferred to NMR tube using a pipette and was measured. The NMR was performed on the dissolved solution on an Avance III HD 500MHz spectrometer. Abbreviated as follows: s= singlet, d = doublet, t = triplet, q = quartet, p = pentet, m = multiplet, br = broad.

Mass Spectroscopy. Mass spectrometry analyses of coronene and K_3coronene were performed in an Agilent QTOF 7200 instrument with CI ionization (methane) and analyzing accurate mass (High resolution mode) in positive mode on air-exposed samples by taking few mg out from the glove box and keeping in a fume hood overnight. A total ion chromatogram (TIC) was performed first to identify the number of different species.

Elemental Analysis. The K contents were measured using a Spectro Ciros ICP_OES radial view instrument. K_3coronene samples were digested using a mixture of concentrated nitric acid (4 mL) and sulfuric acid (1 mL) and diluted using ultrapure water. The C and H contents of the products were identified using a Thermo EA1112 Flash CHNS-O analyzer. Powders of ca. 1

mg were loaded into a Pb capsule, which was sealed mechanically inside a He-filled glovebox. The final compositions were given by averaging results of three samples from the same reaction.

Magnetometry. Magnetic measurements of K_3coronene samples were performed using Quantum Design SQUID, MPMS magnetometer. A known amount of K_3coronene was loaded into custom-made quartz ampules and sealed under high vacuum ($\sim 2 \times 10^{-5}$ Torr). Temperature dependent magnetization, M(T), 2– 300 K, was carried out at an applied field of 10 mT following the Zero Field Cooled (ZFC) and Field Cooled (FC) protocols. Temperature dependent (5 – 300 K) magnetic moment measurements at applied fields of 6.5 T and 5.5 T were performed by initially cooling the sample down to 2K under the applied magnetic field (5.5 T or 6.5 T), followed by M(T) measurement by gradual increase of temperature. Field-dependent magnetization, M(H) isotherms at 2K, 3K, 4K, 5K, 6K, 8K, 10K, 20K, 25K, 30K, 35K, 40K, 45K, 50K, 100K, 150K, 200K, 250K and 300K were performed by continuous sweeping of the applied magnetic field from 0 Oe to 7 T. The M(H) isotherms of K_3cor-1 are given in Figure S24, section S7, SI. The molar mass is taken as 418.82 g/mol (corresponding to $K_{3.03}$coronene as obtained from Rietveld refinement) and the mass taken for K_3cor-1 is 6.3 mg.

Electron Paramagnetic Resonance. For the purpose of electron paramagnetic resonance (EPR) measurements, K_3coronene samples were sealed in 4 mm diameter EPR quartz suprasil Willmad tubes. The continuous wave (cw) EPR measurements were carried out on the Bruker E500 X-band spectrometer, operating at 9.4 GHz and equipped with a Bruker 4122SHQE cylindrical resonator, Oxford Instruments ESR900 cryostat and ITC503 temperature controller, with temperature stability better than ± 0.05 K. The EPR spectra were typically measured at 0.1 mW microwave power, while the modulation field was set to 0.01 mT amplitude and 100 kHz modulation frequency.

Computational Details. 195 PAHs as potential candidates for intercalation were extracted from the ZINC15 database of commercially available compounds.[2] Hierarchical agglomerative clustering was employed to cluster these PAHs based on the calculated distance between their molecular orbital energies (HOMO−1 to LUMO+4) computed using the semi-empirical PM6 method[3] as implemented in Spartan. The linkage function from the scipy.cluster.hierarchy module was used to perform the hierarchical clustering with Ward's minimum variance criterion[4] to minimize the total within-cluster variance. The pairwise distances between data

points were computed using the Euclidean metric. The dendrogram, constructed from the derived linkage matrix, illustrates the hierarchical structure of the PAHs data and allows for the identification of distinct groups with similar electronic structure.

To compute the convex hull of stability for different extents of K intercalation into coronene, hypothetical structures were constructed by inserting K into voids quantified by finding the diameter of the largest included sphere and free sphere using Zeo++[5] in the crystal structure of solid coronene (CSD code: CORONE).[6] Each structure was optimized using plane-wave based density functional theory (DFT) as implemented in VASP.[7] The van der Waals corrected functional, optB86b-vdW,[8] was used throughout, with projector augmented wave potentials,[9] a plane-wave cutoff energy of 520 eV and a *k*-point spacing of 0.2 $Å^{-1}$. Structures were optimized until forces fell below 0.01 eV $Å^{-1}$. The energy difference was calculated as

$$\Delta E = (E(n\ \mathrm{K_x coronene}) - n\ E(\mathrm{coronene}) - n\ \mathrm{x}\ E(\mathrm{K}))\ /\ n$$

where n is the number of coronene molecules in the computational cell, $E(n$ $K_x$coronene) is the energy of the hypothetical intercalated structure, E(coronene) is the energy of a single coronene molecule and E(K) the energy of one atom in K metal. This energy difference is plotted in Figure 1(d) along with a solid line representing the convex hull of stability.

Structure solution: The structure of the main phase of K_3coronene was solved on synchrotron PXRD data for sample K_3cor-4. This dataset was selected as the data with the lowest level of the secondary phase ($K_x$coronene′) which contained refinable peaks which are independent of the main phase, thus allowing for the reliable refinement of scale parameters between the two phases. K_3cor-1 was not used, as it is possible that a small amount of $K_x$coronene′ remains in the sample, but without and refinable reflections which are independent of the main phase, it would not be possible to refine reliable scale parameters for the two phases.

Peak indexing, Structure solution and Rietveld refinement were performed using Topas Academic software.[10] The unit cell of the main phase was indexed by fitting the reflections in the data between 3.8 – 16.5 ° (d-spacing range 12.5 – 2.9 Å), fitting the data with 30 reflections. The unit cell was then indexed using the auto-indexing in Topas, with the best fit corresponding to the unit cell described in the main text. Note that the unit cell was initially indexed to the space group *C2/m*, however, this resulted in unsuccessful attempts at a structure solution and so the symmetry was reduced to *C2* as presented in the main text.

To solve the structure of K_3coronene an initial model for the Rietveld refinement was obtained *via* simulated annealing, as follows.

For the coronene, two sites with randomly generated co-ordinates were used as the centroid for potential coronenes. On each site, two coronene molecules were centred, with a fixed occupancy of 0.5, and the geometric centre of each molecule was fixed to the site. Each of the two molecules on each site were allowed to rotate freely around all three crystallographic axis, independently of each other. Throughout both the structure solution and Rietveld refinement all of the coronene molecules were treated as rigid bodies. For the K sites, the initial model started with 15 separate sites on the four-fold general position, with occupancies allowed to vary freely. The total K content of the cell was constrained by applying a penalty function to the total calculated K content:

$$pen = (6 - nK)^2 \times 1000$$

Where *pen* is the applied penalty to the fitting parameters, and *nK* is the total K content of the current model with 6 matching the expected K content from the nominal composition. Simulated annealing was then run on this model for 2,500,000 refinement steps using the Automatic temperature control in Topas, and the best fitting result becoming the starting point for Rietveld refinements.

During the initial refinements, anti-bumping penalties were introduced between atoms / molecules to restrain the model to be physically reasonable as follows:

For K-K distances, for K sites which have an occupancy of > 0.5 we set these such that they must be > 3 Å from another K atom, and more than 2.4 Å from a coronene. When the occupancy is ≤ 0.5 we removed the distance constraint to other K atoms (provided that they also have a ≤ 0.5 occupancy), and applied the K – coronene distance to only one of the two coronene molecules on each site. For all K sites with an occupancy of > 0.5, anti-bump penalties were applied to all of their interatomic distances, for those ≤ 0.5 anti-bump penalties were applied to at least one of each of the coronene pairs on the neighbouring sites (e.g. K3 – a OR b and K3 - c).

For distances between coronene molecules, an anti-bumping penalty was applied to contacts between coronene molecules between the two sites to be greater than 1.5 Å.

After all of the structural parameters were released, additionally the occupancy of the coronene molecules was allowed to refine, constrained such that the total coronene occupancy on each site was restricted to a total of 1. During these refinements, four of the K sites refined to zero occupancy and were removed from the refinement. One of the coronene sites refined such that both molecules on the site overlapped and so were combined into one, and the occupancy fixed

to be one. This resulted in the final model containing three coronene molecules (two sharing one site, and one on the other site) and eleven K sites.

As part of error checking the result, for each coronene molecule, we assembled the model of the local representation presented in the main text to ensure that local orderings with reasonable interatomic distances were possible. We define this as K – K distances greater than 3 Å, K – coronene (the shortest distance to any part of the coronene molecule) distances greater than 2.4 Å and coronene – coronene contacts being greater than their corresponding intramolecular distances, e.g. C- C distances being greater than 1.5 Å. We note that in the model of the local representation there is one K – coronene distance which is shorter than these restrictions, this is a result of it being one specific local arrangement, which is impossible to constrain within the disordered Rietveld model without reducing the model symmetry to P1.

In the final refinement, all of the structure parameters, background and peak shape parameters were refined. With a final R_{wp} = 3.8% for 111 parameters. To take into account the contribution of the secondary phase which contains a number of hkls which overlap with the main phase, a Pawley fit was performed on our sample which contained the highest quantity of this phase, the resulting hlk-I table was then fixed, and then included in the final refinement of the main crystalline phase, with the only refining parameters being the peak shape and a scale parameter. This inclusion of the secondary phase resulted in the refinement of the main phase being stable and reducing the R_{wp} by ~ 1.5%.

DFT calculations of electronic properties and exchange interactions: For the purpose of calculating the electronic properties and exchange interactions, density functional theory calculations (DFT) were carried out using the ORCA code.[11, 12] For the structure optimization followed by vibration frequency calculations, B3LYP[13] method handled with 6-31G**[14-20] basis set was used. For the charged coronene molecules, calculations of EPR parameters for C and H atoms were performed using B3LYP and EPR-II[21] basis set after the optimization. The default ORCA setting was used for the other parameters. Broken-Symmetry (BS) DFT calculations implemented in ORCA were carried out to calculate exchange coupling between two charged (total charge 2− and 6−) coronene molecules by localizing one or three unpaired electrons on each coronene molecule, respectively. B3LYP functional and 6-31G** basis set were used. For the BS-DFT, highly symmetric coronene structure (with the bond lengths of 1.08 Å for C-H and 1.4 Å for C-C) was also used as input files.

Section S2. Structure and porosity analysis of different PAHs

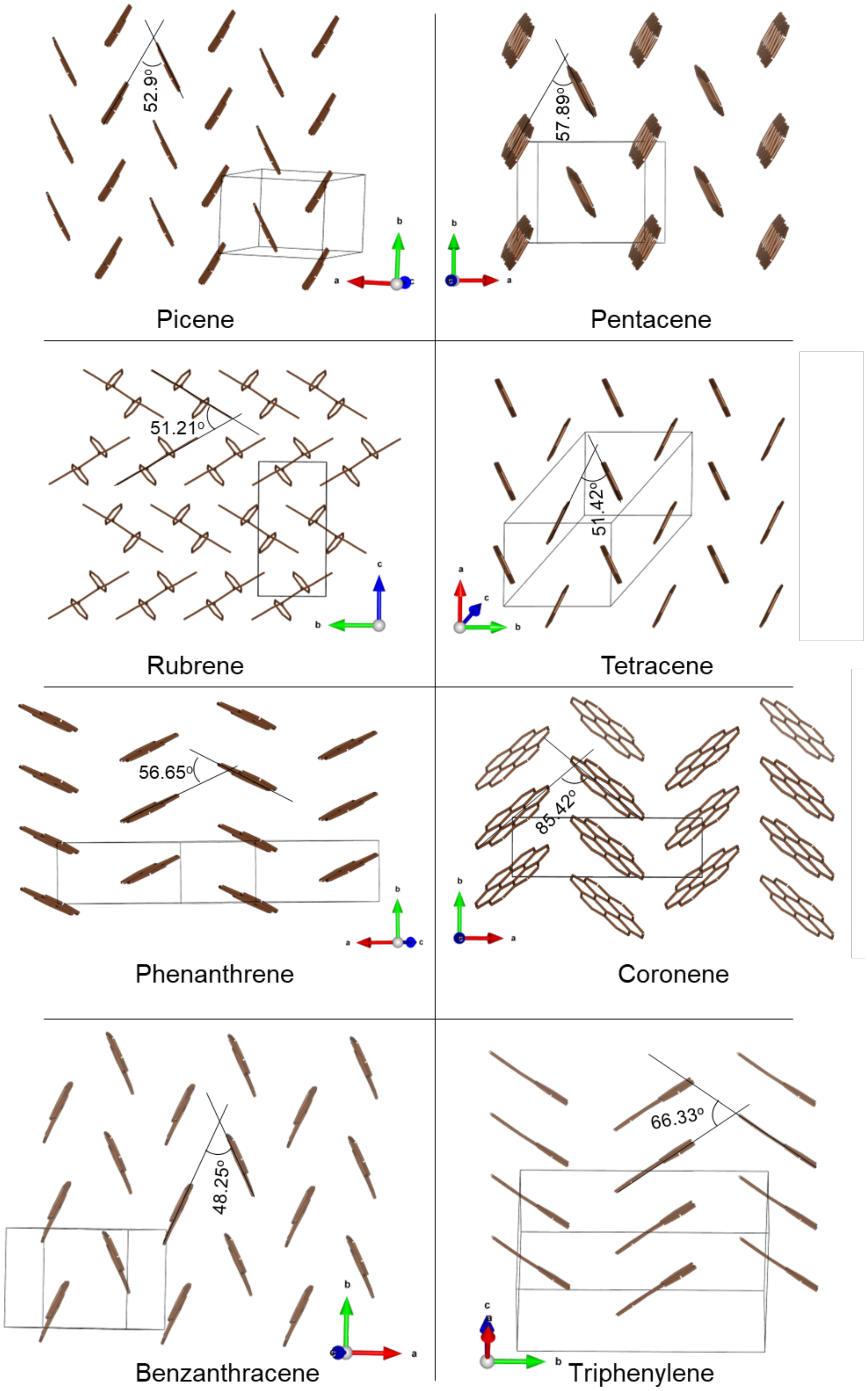

Figure S1. Crystal structure of different PAHs showing the herringbone molecular packing.

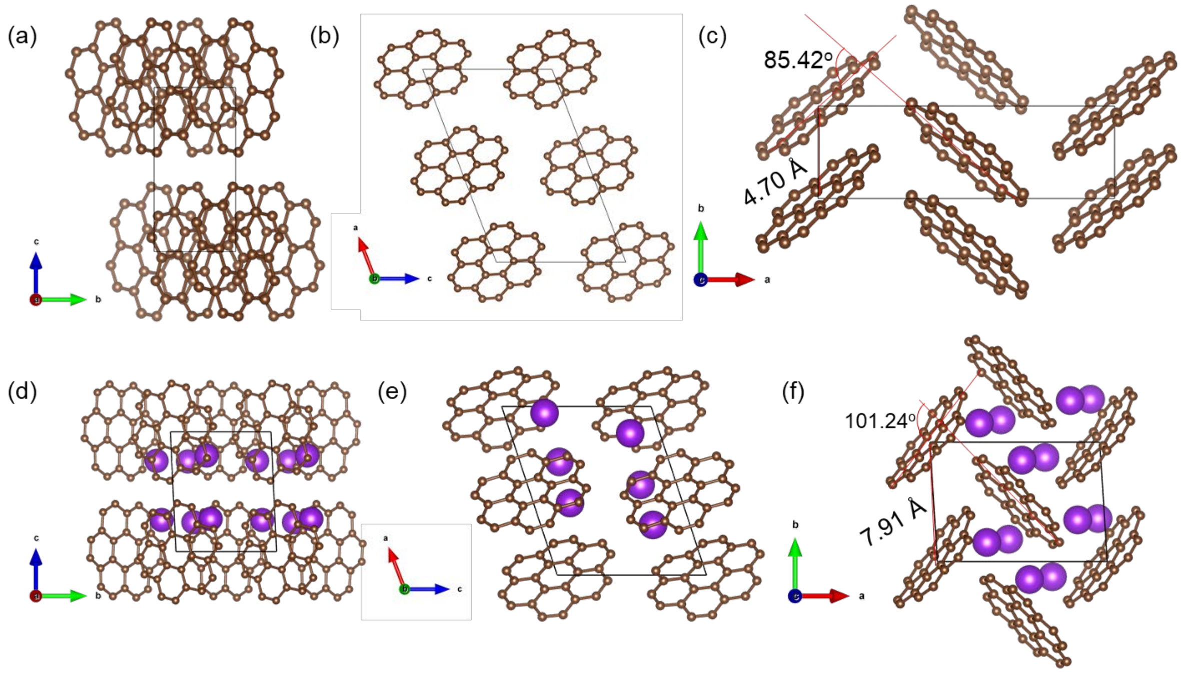

Figure S2. (a-c) Crystal structure of pristine coronene along *a*-, *b*- and *c*- directions, respectively. (d-f) Computed structure of the lowest energy K_3coronene along *a*-, *b*- and *c*- directions, respectively. The K^+ ions intercalation is possible only after a substantial rearrangement of the coronene molecules and their intermolecular distances. Purple balls represent the K^+ ions.

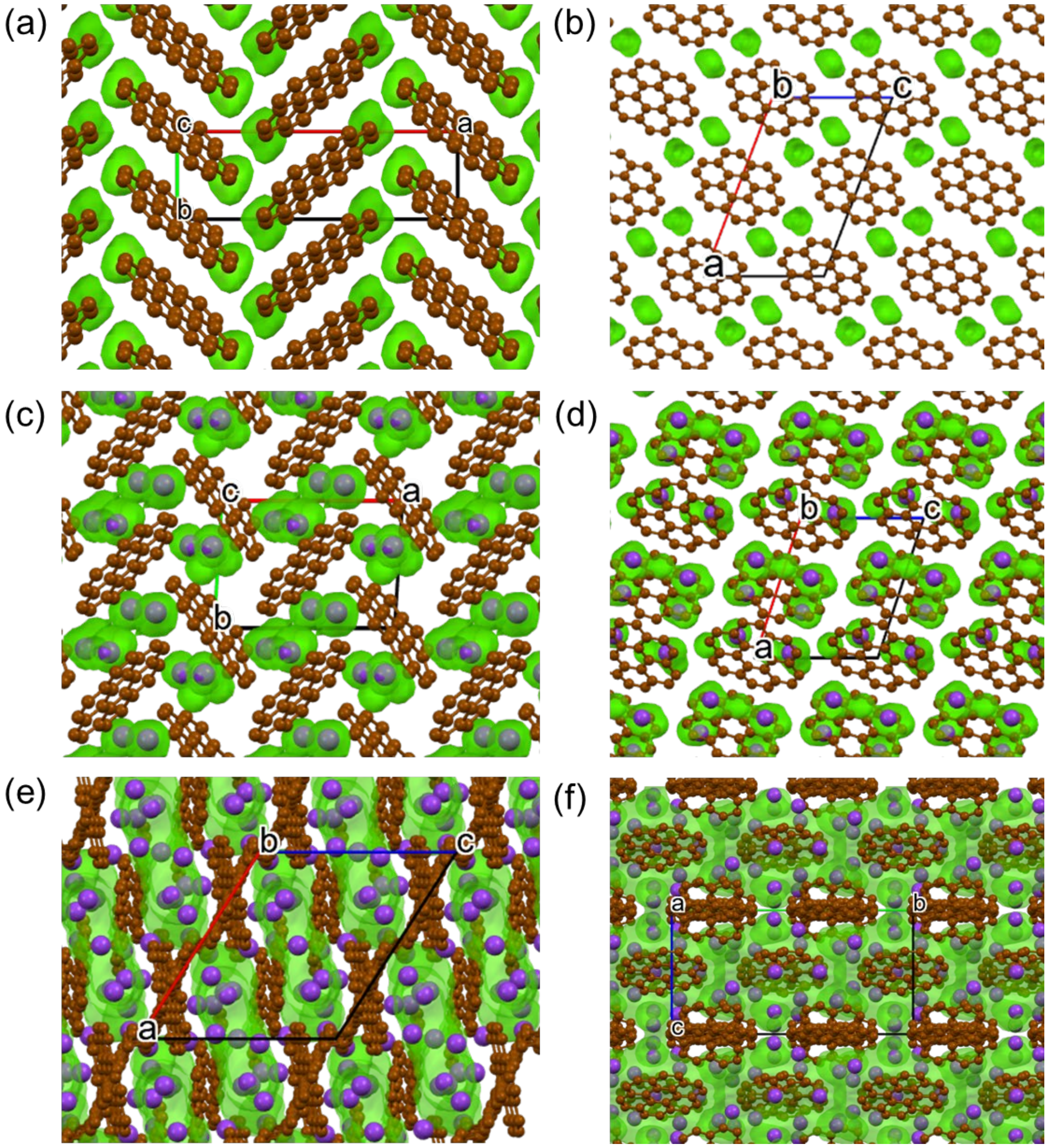

Figure S3. (a, b) Void spaces (in green) in pristine coronene present in between the layers computed with a probe radius of 0.65 Å. (c-d) Void spaces (in green) in the lowest energy structure of K_3coronene calculated using Crystal Structure Prediction (CSP) software computed with a probe radius of 1.00 Å. (e, f) Void spaces in the model of the local representation of the refined experimental structure of K_3coronene computed with a probe radius of 1.00 Å. The void spaces are computed and plotted using the software Mercury[22]. Brown and purple balls represent carbon and potassium respectively.

Porosity analysis

Table S1. Porosity of molecular crystals known to form intercalated compounds with potassium through solid state reaction with KH and their comparison to crystalline coronene. Porosity analysis is performed using Zeo++.[5] Room temperature crystal structures are taken from the Cambridge Structural Database[23] using the given codes.

**Molecule**	**CSD Code**	**Diameter of largest included sphere (Å)**	**Diameter of free sphere (Å)**
Tetracene	TETCEN03	2.22	1.20
Pentacene	PENCEN19	2.17	1.19
Picene	ZZZOYC04	2.31	1.19
Rubrene	QQQCIG40	2.22	1.31
Coronene	CORONE11	2.24	1.35

Section S3. Variable Temperature and variable composition synthesis of K-coronene.

3.1 Variable temperature powder X-ray diffraction (VT-PXRD) measurement of KH with coronene.

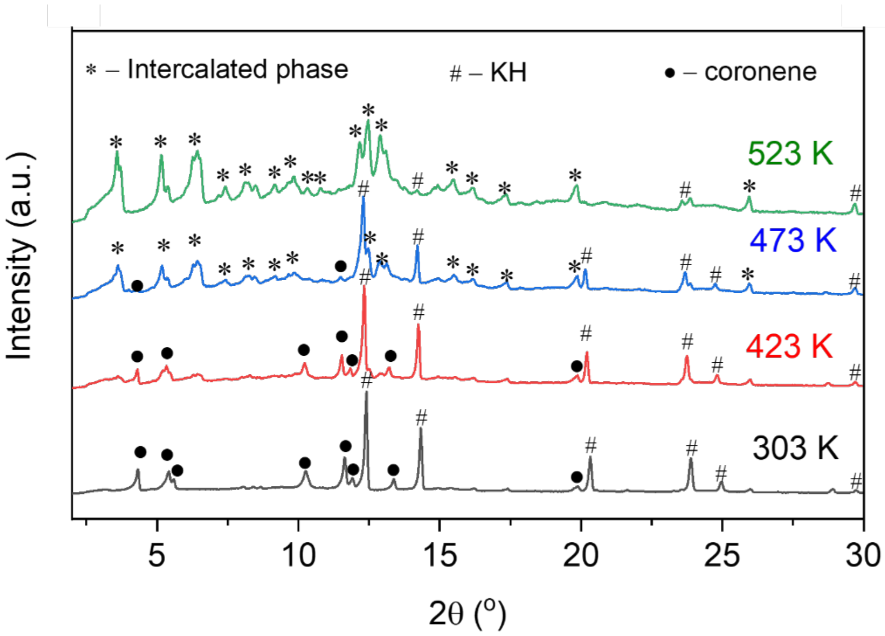

Figure S4. VT-PXRD of KH and coronene (3:1 ratio) with a dwelling period of 1 h at each temperature. Peaks marked by * are new, demonstrating the formation of new intercalated phase. Peaks from the starting materials coronene (marked by •) and KH (◆) are shown as well. The measurements are done in a Rigaku diffractometer with Mo source (λ ~ 0.71 Å) with an Anton Parr HTK 1200N high temperature stage.

3:1 molar ratio of KH and coronene were ground using a pestle and mortar in an argon filled glove box and a protocol is used for VT-PXRD measurements as described in the methods (Section S1). The reaction is monitored at temperatures of 303 K, 423 K, 473 K and 523 K. At 423 K and below, we mainly observe peaks for the starting materials coronene and KH, indicating the intercalation of K in coronene does not occur at low temperatures. Once the temperature is ramped to 473 K, we observe the formation of crystalline intercalated phase, marked by *. These peaks are found to be different as compared to the peaks observed from the VT-PXRD of K-metal and coronene. The new phase co-exists with substantial amount of starting material, KH and coronene. At 523 K, we observe the intercalated crystalline phase as the major phase, with minor amounts of KH present (Figure S4). From VT-PXRD, we chose 523 K as the optimum temperature for K intercalated coronene.

3.2 Variable composition synthesis of KH with coronene.

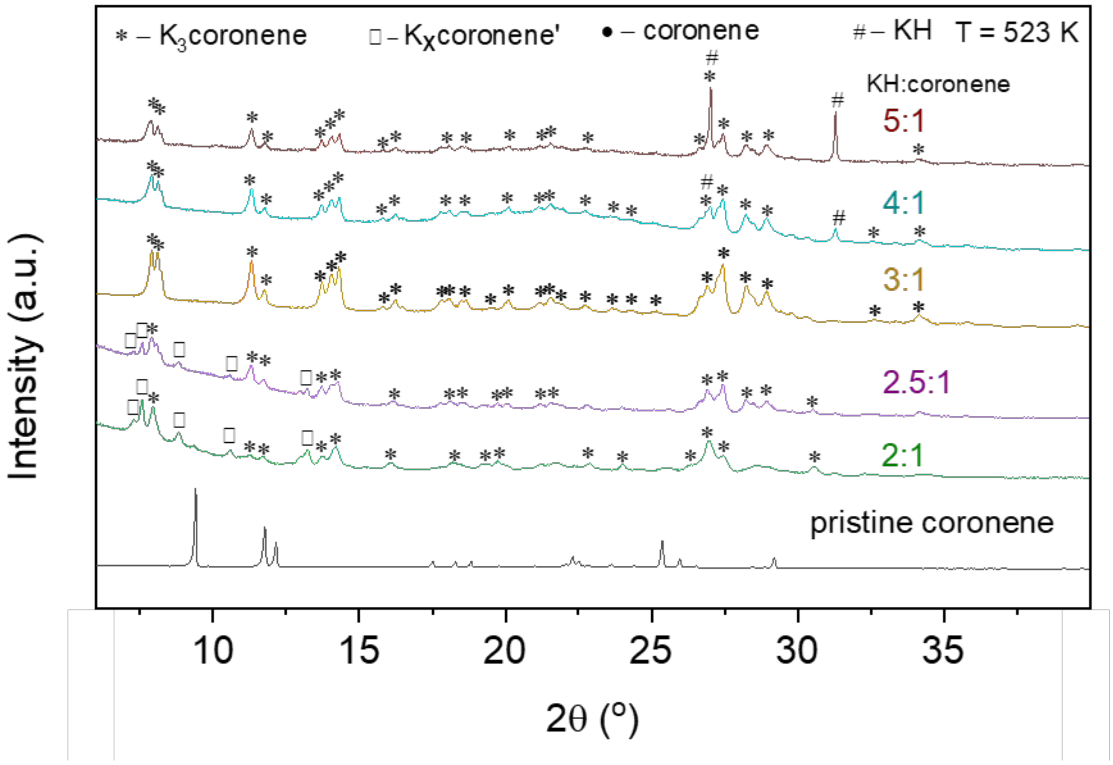

Figure S5. Variable composition reaction between KH and coronene at reaction temperature, T = 523 K for times described in the text on page S14. The coronene peaks are marked by •, KH peaks by #, known intercalated phase, $K_{3.03}$coronene by * and unknown intercalated phase, $K_x$coronene' are marked by □. The measurements are done in a Bruker diffractometer at room temperature having Cu source (λ ~ 1.54 Å). The products are labelled using the format "K-source – K-source:coronene ratio – temperature".

KH and coronene are ground at stoichiometries of 2:1, 2.5:1, 3:1, 4:1 and 5:1 inside an argon filled glove box and sealed in a 5mm inner diameter quartz tube and heated to 523 K and dwelled for 6 h. At lower stoichiometric ratio of KH:coronene (2:1 or 2.5:1), we observe significant amount of the secondary crystalline phase ($K_x$coronene′, marked by □) along with the main phase (K_3coronene, marked by *). As the ratio of KH:coronene is increased from 2:1 to 2.5: and to 3:1 the intensity of $K_x$coronene′ peaks are found to decrease relative to K_3coronene peaks, and for 3:1 ratio of KH:coronene, we mainly observe $K_{3.03}$coronene as the major phase with little or no $K_x$coronene′ peaks present (Figure S5). Higher ratios of KH:coronene (4:1 and 5:1) leads to the formation of $K_{3.03}$coronene along with excess KH peaks, indicating a nominal composition of 3:1 is the best ratio for obtaining a new intercalated material.

For 2:1 ratio of KH:coronene (KH-2-523), the starting materials are ground in argon filled glove box, vacuum sealed (~ 2 × 10^{-5} Torr) in 5 mm inner diameter quartz tubes, heated to 523 K with 12 h dwelling. The compound obtained is then reground, sealed and heated to 523 K with 6 h dwelling. For 2.5:1 ratio of KH:coronene (KH-2.5-523), the starting materials are ground in argon filled glove box, vacuum sealed (~ 2 × 10^{-5} Torr) in 5 mm inner diameter quartz tubes, heated to 523 K with 18 h dwelling. The compound obtained is then reground, sealed and heated to 523 K with 6 h dwelling. For 4:1 and 5:1 ratio of KH:coronene (KH-4-523 and KH-5-523), the starting materials are ground in argon filled glove box, vacuum sealed (~ 2 × 10^{-5} Torr) in 5 mm inner diameter quartz tubes, heated to 523 K with 6 h dwelling. 3:1 ratio of KH:coronene synthesis is given in methods section (see section S1).

3.3 Variable temperature powder X-ray diffraction (VT-PXRD) measurement of K metal with coronene.

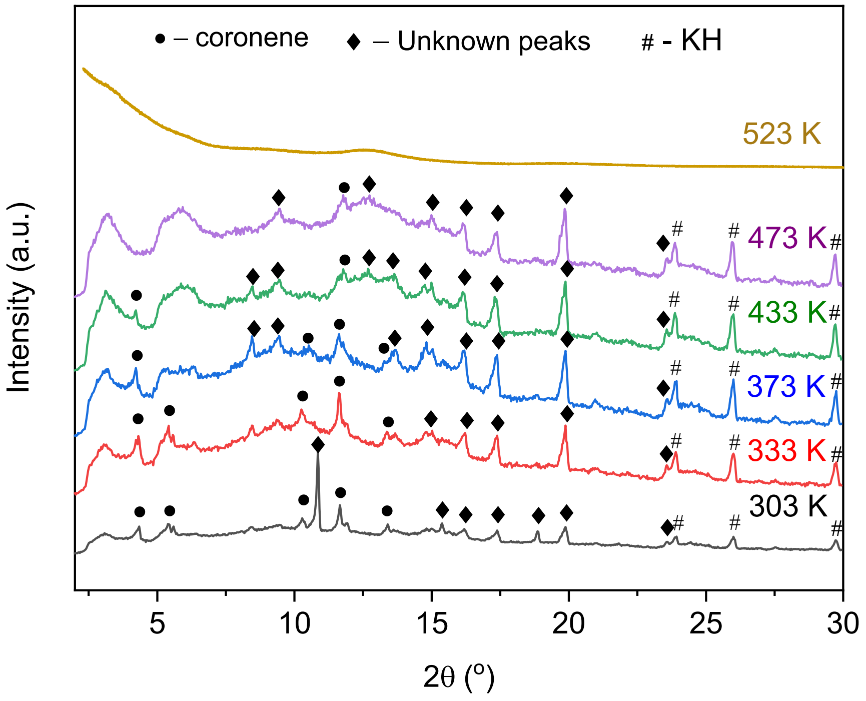

Figure S6. VT-PXRD of K-metal and coronene (3:1 ratio) with a dwelling period of 1 h at each temperature. Peaks marked by ◆ are unknown peaks, indicating formation of new intercalated phase(s) of K-coronene along with KH (marked by #). Peaks marked by • are peaks form the starting material coronene. The measurements are done in a Rigaku

diffractometer with Mo source (λ ~ 0.71 Å) with an Anton Parr HTK 1200N high temperature stage.

3:1 molar ratio of K metal and coronene were ground in an argon filled glove box and filled in a quartz capillary of 1.5 mm inner diameter for VT-PXRD measurements. The capillary was then mounted in a Rigaku powder X-ray diffractometer containing a Mo source (λ ~ 0.7107 Å) and heated using an Anton Parr HTK 1200N high temperature stage. The reaction is monitored at temperatures of 303 K, 333 K, 373K, 433 K, 473 K. The temperature is then ramped and dwelled at this temperature for 1 h. PXRD measurement were performed in the two-theta range of 2° – 30° to monitor the reaction progress at each temperature. Reaction between K metal and coronene (•) was found to mainly yield amorphous products, along with unknown crystalline phase(s) (marked by ◆) and KH (marked by #) (Figure S6). The reaction at 523 K is done by grinding 3:1 ratio of K-metal and coronene, vacuum sealing in a 5 mm quartz tube, ramping it to 523 K followed by dwelling for 6 hours.

3.7 Variable composition synthesis of K metal with coronene

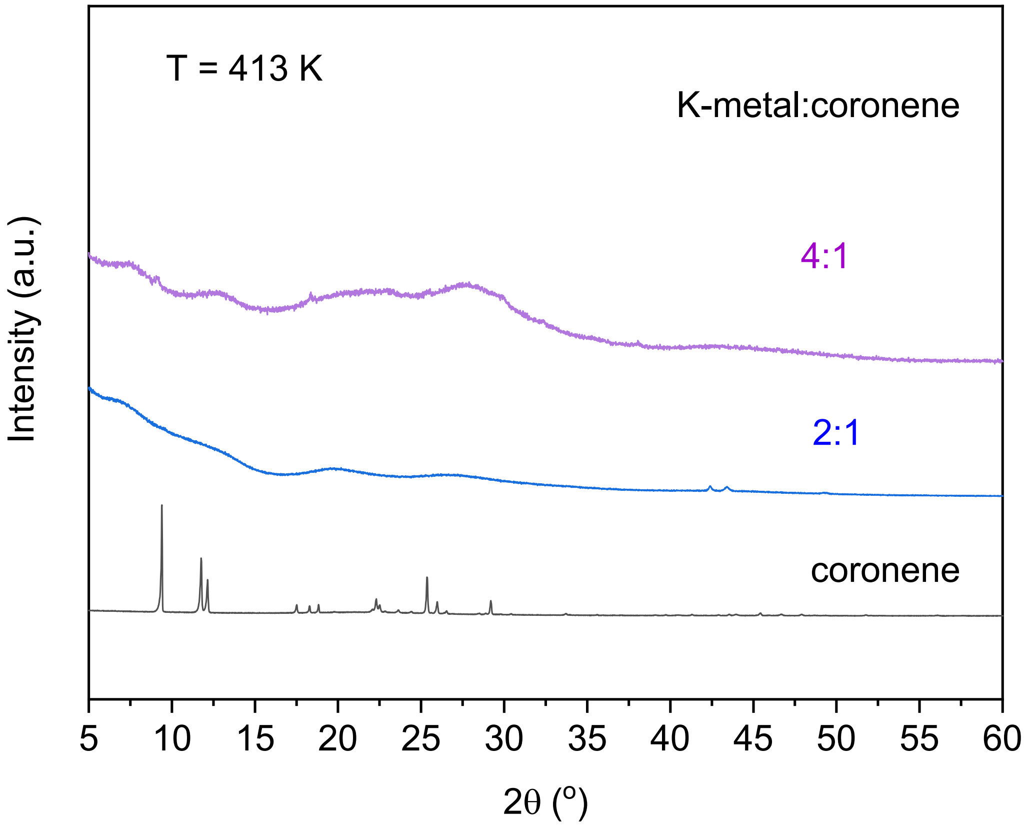

Figure S7. Variable composition reaction between K-metal and coronene. The coronene peaks are marked by • and intercalated phase is marked by □. The measurements are done in a Bruker diffractometer at room temperature having Cu source (λ ~ 1.54 Å).

K metal and coronene were taken in other stoichiometric ratio of 2:1 and 4:1 inside an argon filled glove-box and ground for 10 mins. The reagents are then packed in quartz ampoules having an inner diameter of 5 mm and sealed in high vacuum of ~ 10^{-5} Torr for reactions at 413K with a ramping rate of 2 K/min. 2:1 ratio of K-metal:coronene, heated to 413 K with 6 h dwelling, produces amorphous phase. Similarly higher ratio of 4:1 between K-metal:coronene, heated to 413 K with 12 h dwelling, also produces broad signatures in the PXRD indicating amorphorisation.

Section S4. Elemental Analysis, relative impurity phase and Amorphous content.

Table S2. Mass ratio of starting materials, Elemental analysis, Relative crystalline impurity phase ($K_x$coronene′) from PXRD and % amorphous phase (in weight%) for the different batches of K_3coronene. Magnetic properties were measured on sample K_3cor-1.

**Measurement**	**K_3cor-1**	**K_3cor-2**	**K_3cor-3**	**K_3cor-4**	**K_3cor-5**
KH:coronene amounts taken (mg, ±0.2 mg)	28.6:71.4	36:90	36:90	36:90	36:90
Composition from CHN and ICP (Nominal composition $K_{2.97\text{-}3.02}(C_{24}H_{12})$, with range based on estimated weighing errors)	$K_{3.1\pm0.2}$ $C_{23.6\pm0.2}$ $H_{13.7\pm2.0}$	$K_{3.05\pm0.1}$ $C_{24.0\pm0.2}$ $H_{14.1\pm1.0}$	$K_{3.18\pm0.1}$ $C_{23.8\pm0.2}$ $H_{13.4\pm1.0}$	$K_{3.04\pm0.1}$ $C_{23.9\pm0.2}$ $H_{13.4\pm1.0}$	$K_{2.88\pm0.1}$ $C_{24.5\pm0.2}$ $H_{13.8\pm2.0}$
Relative crystalline impurity phase ($K_x$coronene′) from PXRD	0	1	1.78	2.58	4.64
% amorphous content (weight %)	7.9	0	14.2	27.4	20.0

Section S5. Mass spectroscopy and ^{1}H-NMR.

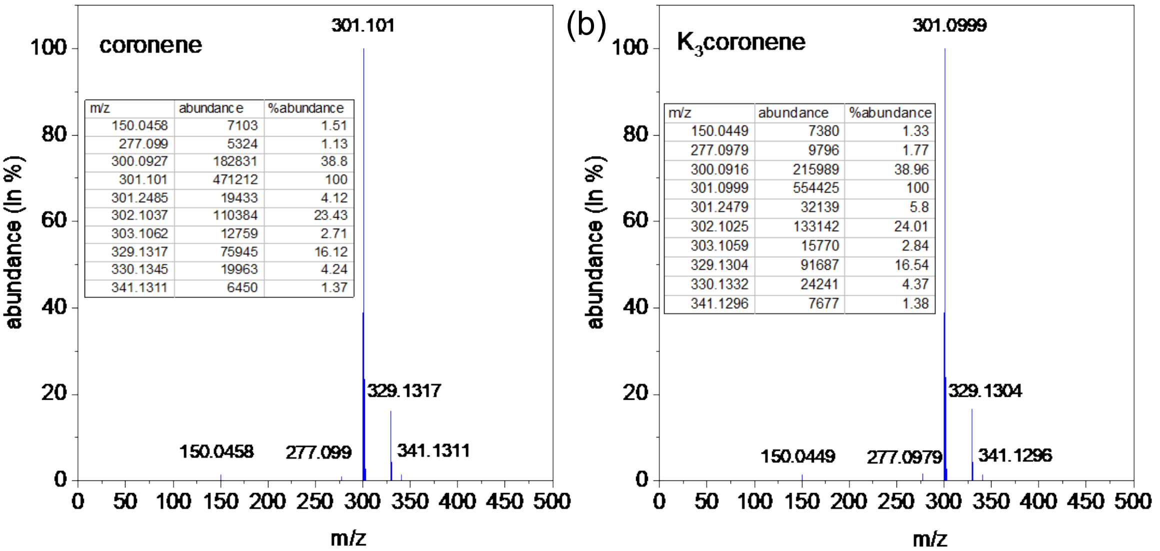

m/z	abundance	%abundance
150.0458	7103	1.51
277.099	5324	1.13
300.0927	182831	38.8
301.101	471212	100
301.2485	19433	4.12
302.1037	110384	23.43
303.1062	12759	2.71
329.1317	75945	16.12
330.1345	19963	4.24
341.1311	6450	1.37

m/z	abundance	%abundance
150.0449	7380	1.33
277.0979	9796	1.77
300.0916	215989	38.96
301.0999	554425	100
301.2479	32139	5.8
302.1025	133142	24.01
303.1059	15770	2.84
329.1304	91687	16.54
330.1332	24241	4.37
341.1296	7677	1.38

Figure S8. Mass Spectra of (a) coronene and (b) K_3coronene. The table inset provides the abundance at each m/z ratio.

Mass Spectroscopy (MS) of the starting material coronene shows a prominent peak at 301.1 m/z (mass to charge ratio) which corresponds to $C_{24}H_{13}^{+}$ (coronene ($C_{24}H_{12}$) molecular weight: 300.09 g/mol). Apart from the main peak, there are minute peaks at 341.1 m/z, 329.1 m/z and 277.09 m/z which are due to coronene + $C_3H_5^{+}$ peak, coronene + $C_2H_5^{+}$ peak and an impurity anthanthrene ($C_{22}H_{12}$; molecular weight: 276.1 g/mol), respectively. MS of K_3coronene shows identical peaks at 301.1 m/z, 341.1 m/z, 329.1 m/z and 277.09 m/z, meaning the chemical species present in K_3coronene and coronene are the same and coronene does not break into smaller molecular fragments during the synthesis (Figure S8).

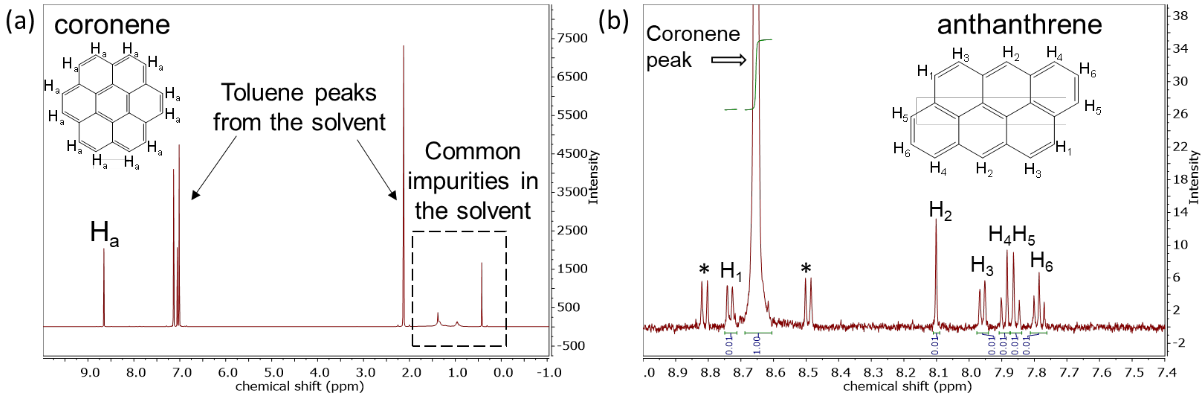

Figure S9. (a) ^{1}H-NMR of coronene in toluene-d^{8} solvent. The peak at 8.65 ppm corresponds to coronene ($C_{24}H_{12}$). (b) Enlarged ^{1}H-NMR (7.4 – 9.0 ppm) showing the impurity peaks H_1 – H_6 corresponding to anthanthrene ($C_{22}H_{12}$). The peaks marked in * are the satellite peaks of the coronene peak.

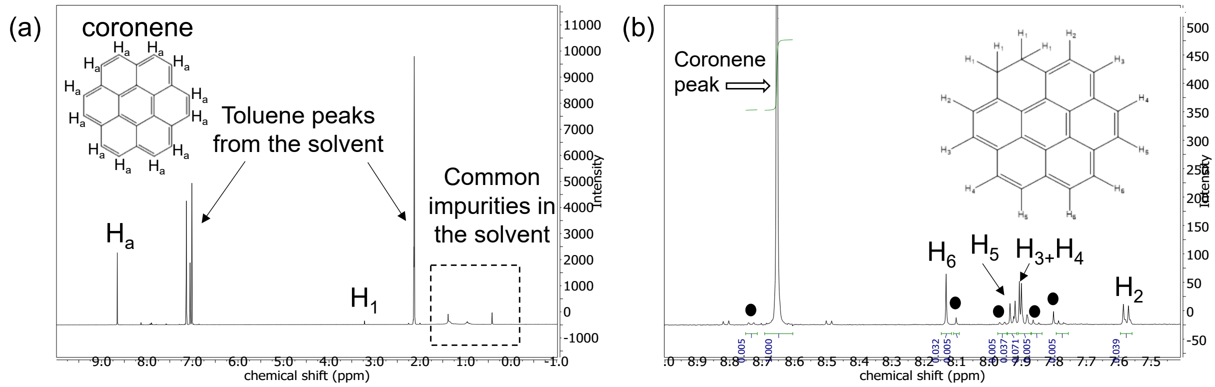

Figure S10. (a) ^{1}H-NMR of K_3coronene in toluene-d^8 solvent. The peak at 8.65 ppm corresponds to coronene ($C_{24}H_{12}$). The peak marked H_1 is from 1,2 dihydrocoronene. (b) Enlarged ^{1}H-NMR (7.4 – 9.0 ppm) showing the peaks H_2 – H_6 corresponding to 1,2 dihydrocoronene ($C_{24}H_{14}$). Peaks maked by • corresponds to anthracene peaks that already present in the coronene starting material.

^{1}H-NMR is done by controlled oxidation of K_3coronene and coronene in air followed by dissolving them in toluene-d^8 solvent. Coronene being a non-polar compound is found to be sparsely soluble in the solvent. Coronene only contains one type of H, and therefore shows a singlet at chemical shift (δ) = 8.65 ppm. Apart from the peak at 8.65 ppm, we observe several low intensity peaks at 8.72 ppm (d), 8.10 ppm (s), 7.96 ppm (d), 7.88 ppm (d), 7.86 (d) and 7.78 ppm (t) belonging to the impurity anthanthrene, that is present in the as-used coronene (Figure S9b). ^{1}H-NMR of oxidised K_3coronene, similarly shows the most intense peak at 8.65 ppm, indicating coronene remains largely intact during the synthetic process. We also observe several other minor peaks apart from the peaks corresponding to coronene and anthanthrene (marked by •). These peaks are situated between 8.2 ppm and 7.5 ppm and one singlet at 3.21 ppm (Figure S10, marked by H_1 - H_6) and is due to hydrogenation of coronene to yield 1,2 dihydrocoronene (Inset of Figure S10b).

The 4 H_1 atoms in 1,2 dihydrocoronene are linked to sp^3 C (inset of Figure S10b), indicating the peak at 3.21 ppm corresponds to H_1 atoms. H_2 – H_5 will lead to doublet with half the intensity compared to H_1, as they contain two H atoms each (These are observed at 7.57 – 7.92 ppm). Similarly, H_6 will lead to singlet with half the intensity of H_1 (This is observed at 8.13 ppm) (Figure S10b). Comparing with the intensity of the extra peaks (Table S3), the peak at 3.13 ppm has twice the intensity as compared to the other extra peaks we observe, confirming the formation of 1,2 dihydrocoronene as a minor side product along with the main intercalated

compound. The total amount of both the impurity (anthanthrene, which is present in the starting material) and the hydrogenation by-product (possibly only formed during the NMR sample preparation as not observed in the MS) is minute given the low intensity of the extra peaks in the NMR spectrum and their minor relative abundance in MS.

Table S3. Integral values of peaks marked H_1 – H_6 in Figure S10. Coronene peak is normalized to 1.

**Hydrogen label**	**Chemical shift (ppm)**	**Integral values**
H_1	3.13	0.069
H_2	7.57	0.039
$H_3 + H_4$	7.90	0.071
H_5	7.92	0.037
H_6	8.13	0.032

Section S6. Crystal structure of K_3coronene

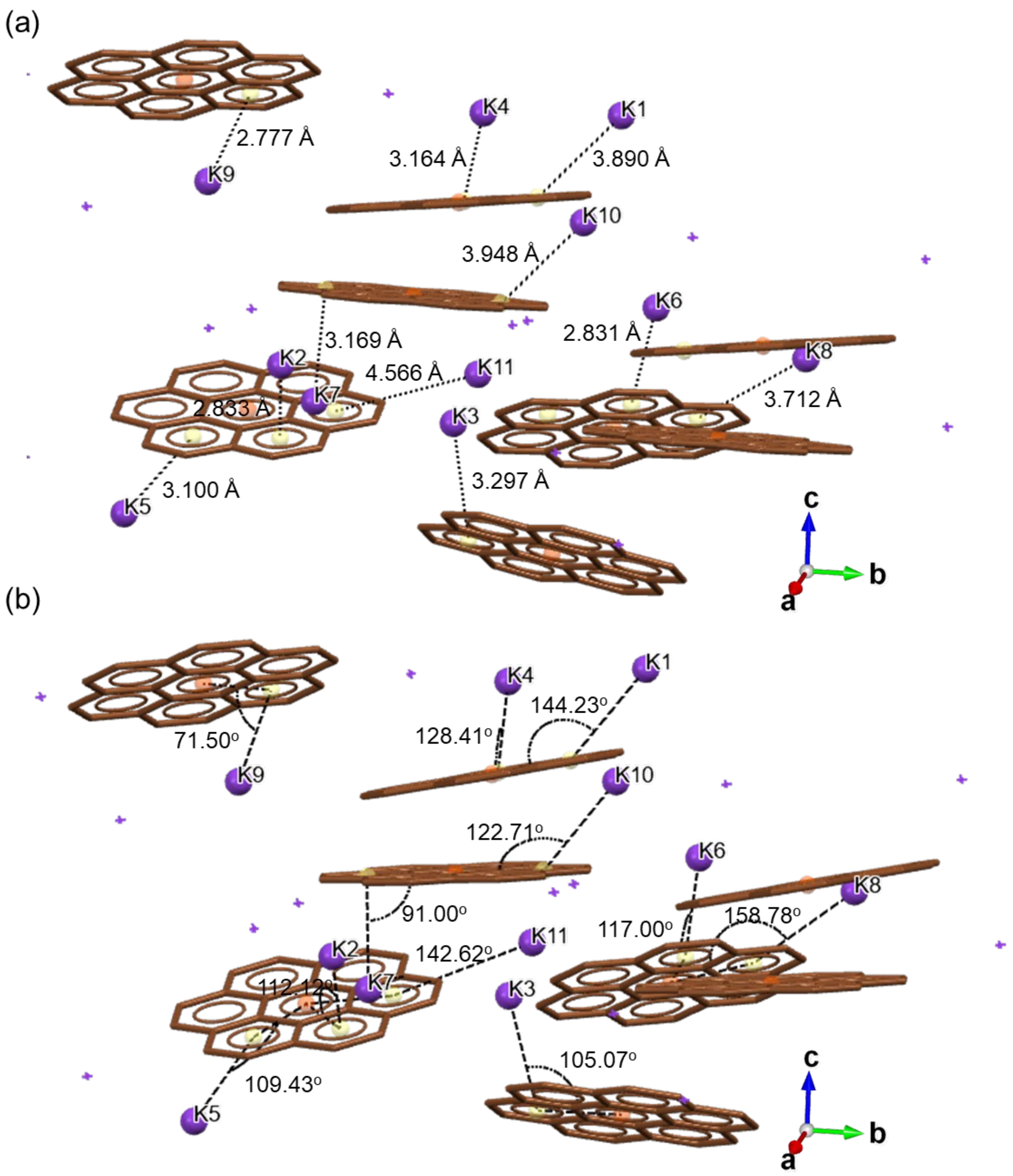

Figure S11. K – coronene distances (in Å) (a) and K – coronene angles (in °) (b), corresponding to K and the centroid nearest benzene ring of the coronene molecules.

Table S4. Distances and angle between the K sites corresponding to the centre of the closest aromatic ring of coronene molecules.

**K Labels**	**Distance (Å)**	**Angle (°)**
K1	3.890	144.23
K2	2.833	112.12
K3	3.297	105.07
K4	3.164	128.41
K5	3.100	109.43
K6	2.831	117.00
K7	3.169	91.00
K8	3.712	158.78
K9	2.777	71.50
K10	3.948	122.71
K11	4.566	142.62

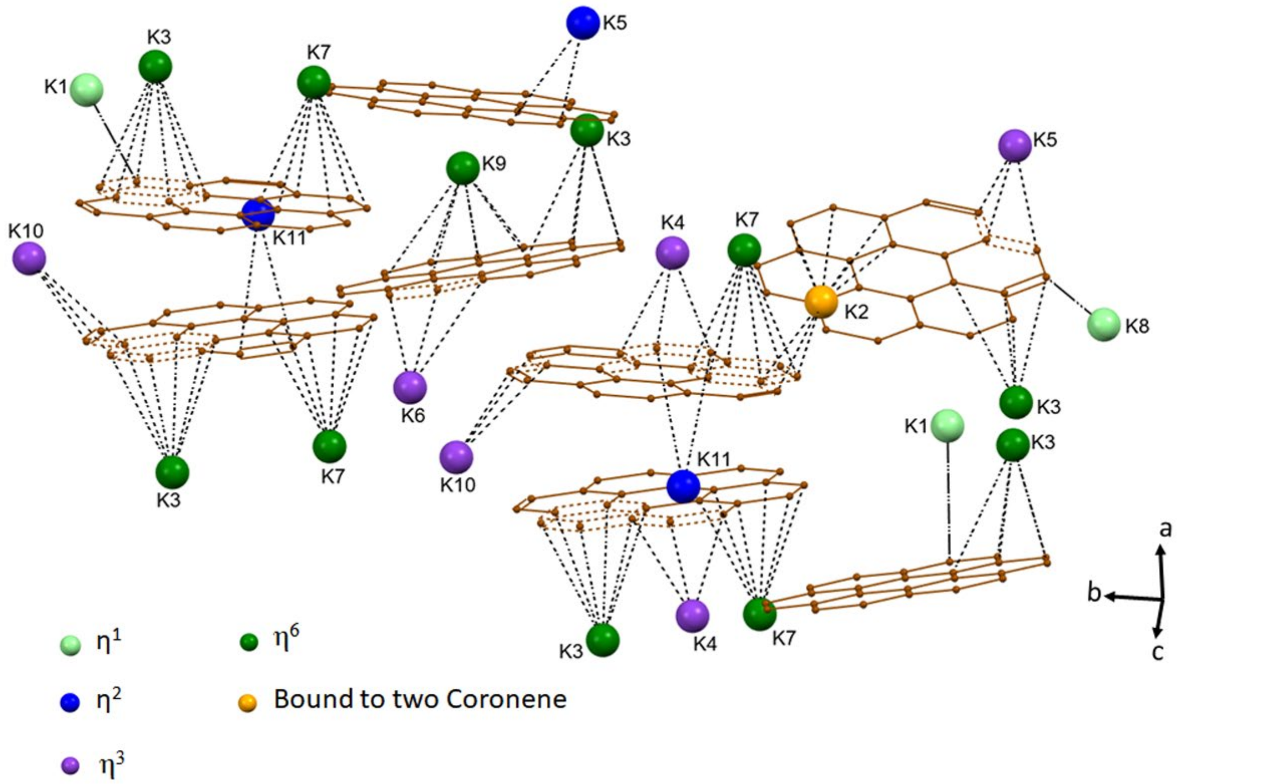

Figure S12. Coordination environments of K in K_3Coronene (model of the local representation) black dotted lines represent the main K–C interactions.

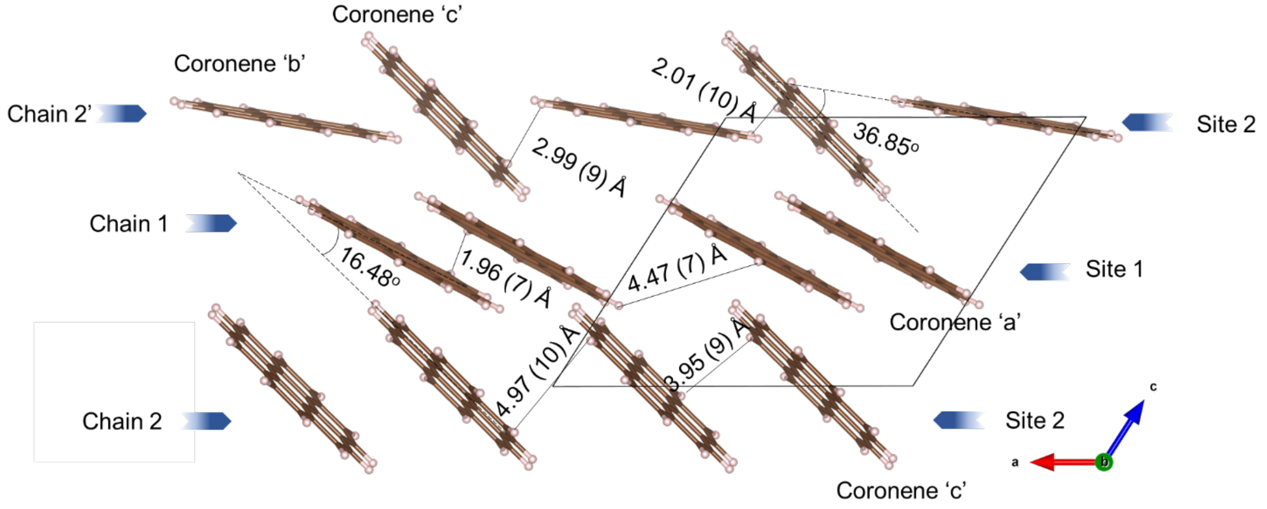

Figure S13. Closest coronene – coronene distances across the three chains. The angles between the coronene molecules are given as well.

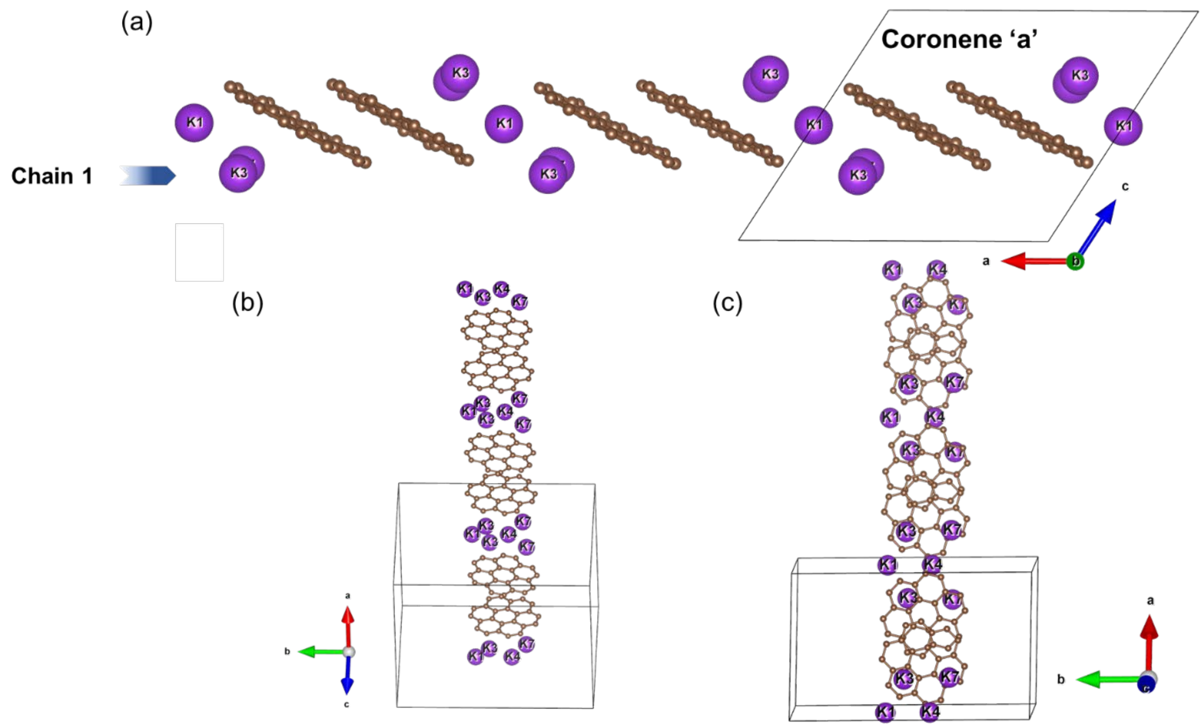

Figure S14. Intercalated K^+ ions (K1, K3, K4, K7) spaced every two coronene 'a' molecules apart in Chain 1. Purple spheres represent K^+ ions within the chain.

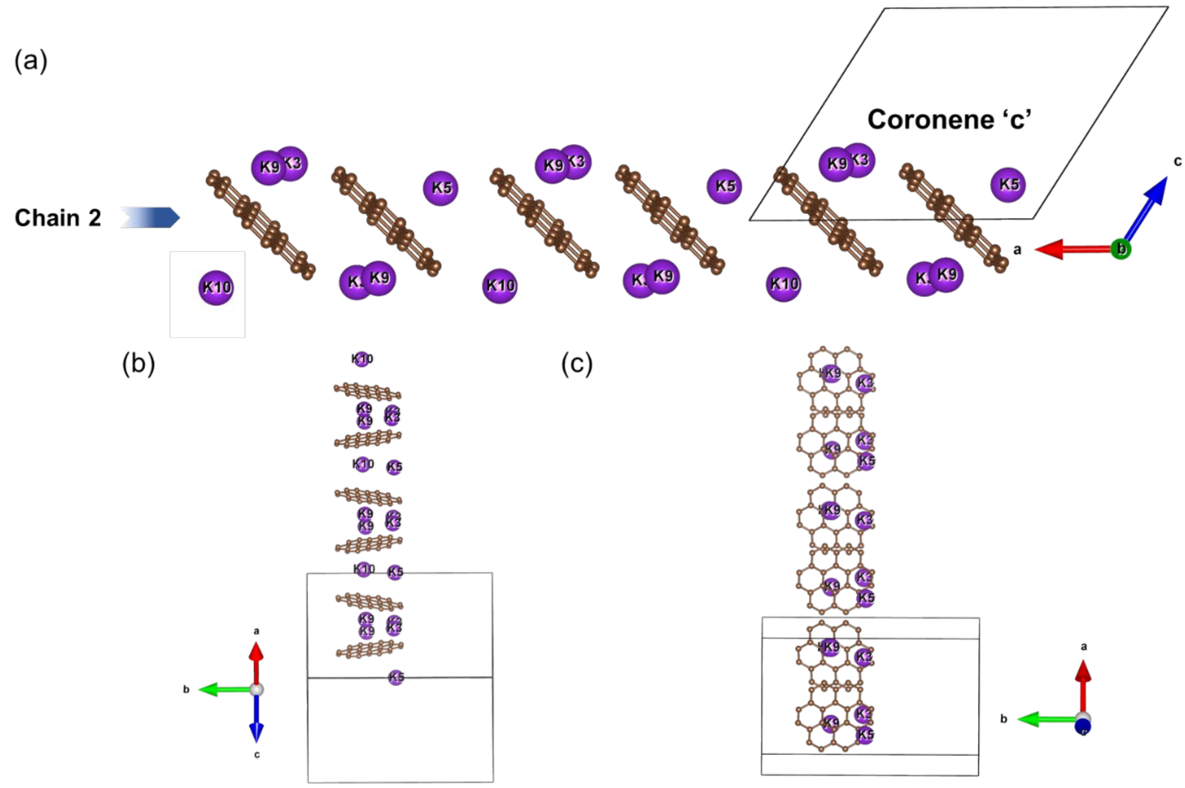

Figure S15. K^+ ions (K3, K5, K9, K10) intercalated between the coronene 'c' molecules in Chain 2. Purple spheres represent K^+ ions within the chain.

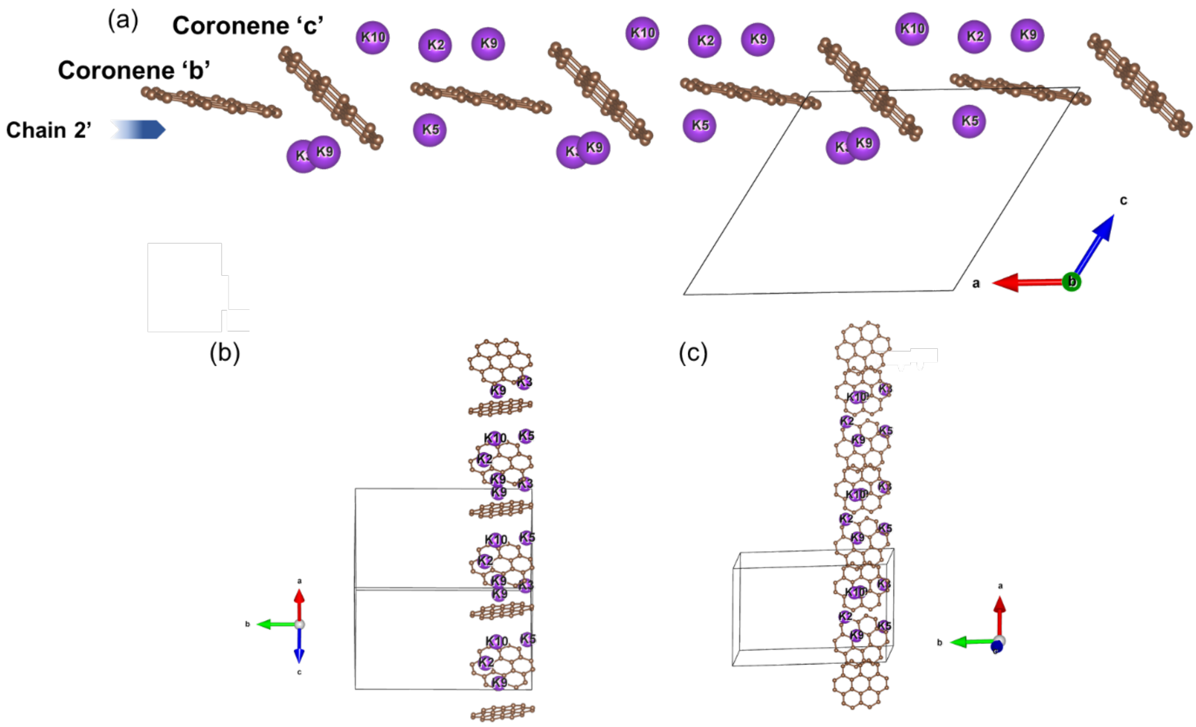

Figure S16. Chain 2′ comprising of alternate arrangement of coronene 'b' and coronene 'c' with K^+ ions (K2, K3, K5, K9, K10) intercalated between them. Purple spheres represent K^+ ions within the chain.

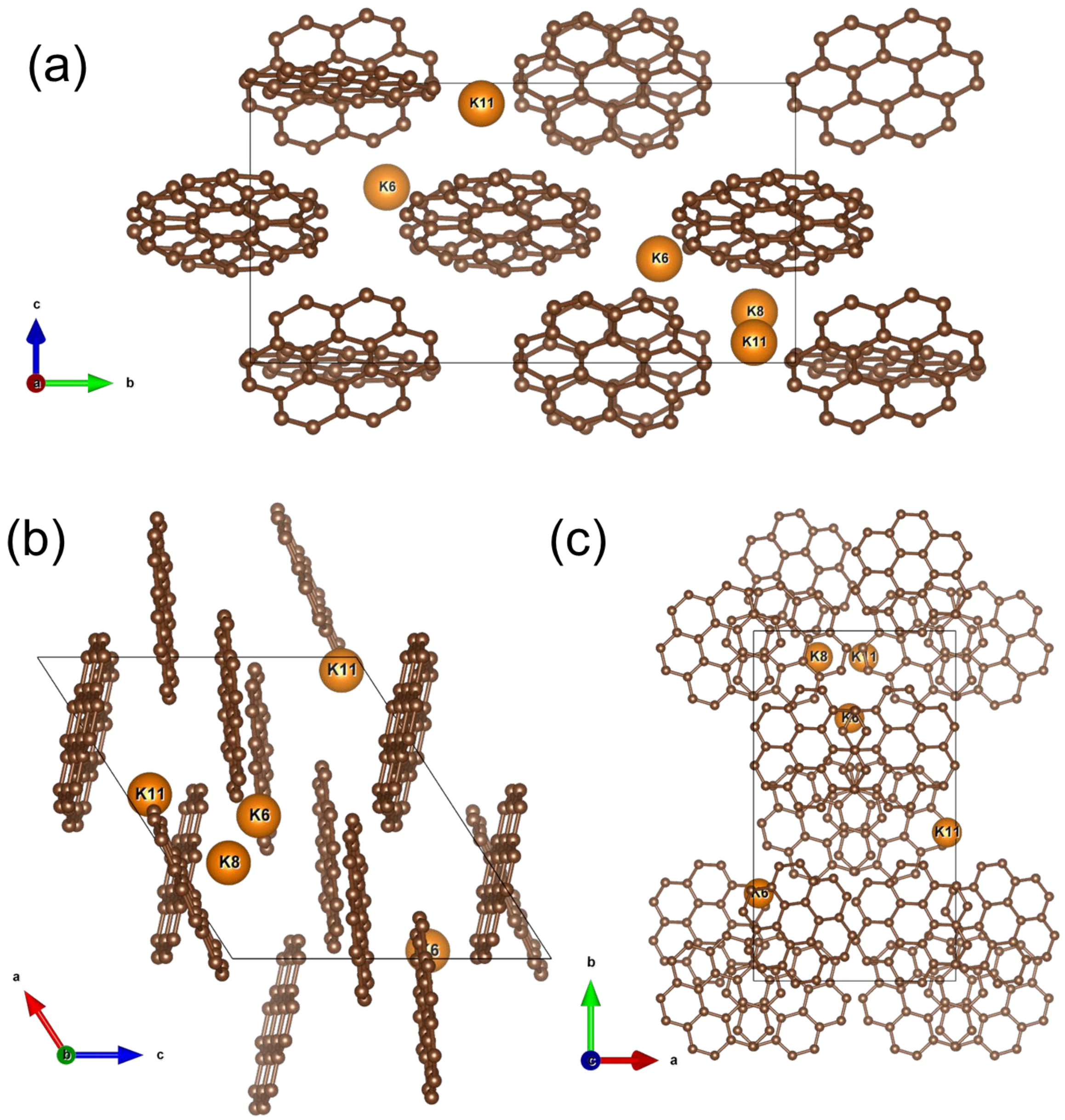

Figure S17. (a-c) Model of the local representation of K_3coronene showing only K^+ ions (K6, K8 and K11) present in between the chains of coronene along *a*-, *b*- and *c*- directions, respectively. Orange and brown balls represent potassium and carbon respectively.

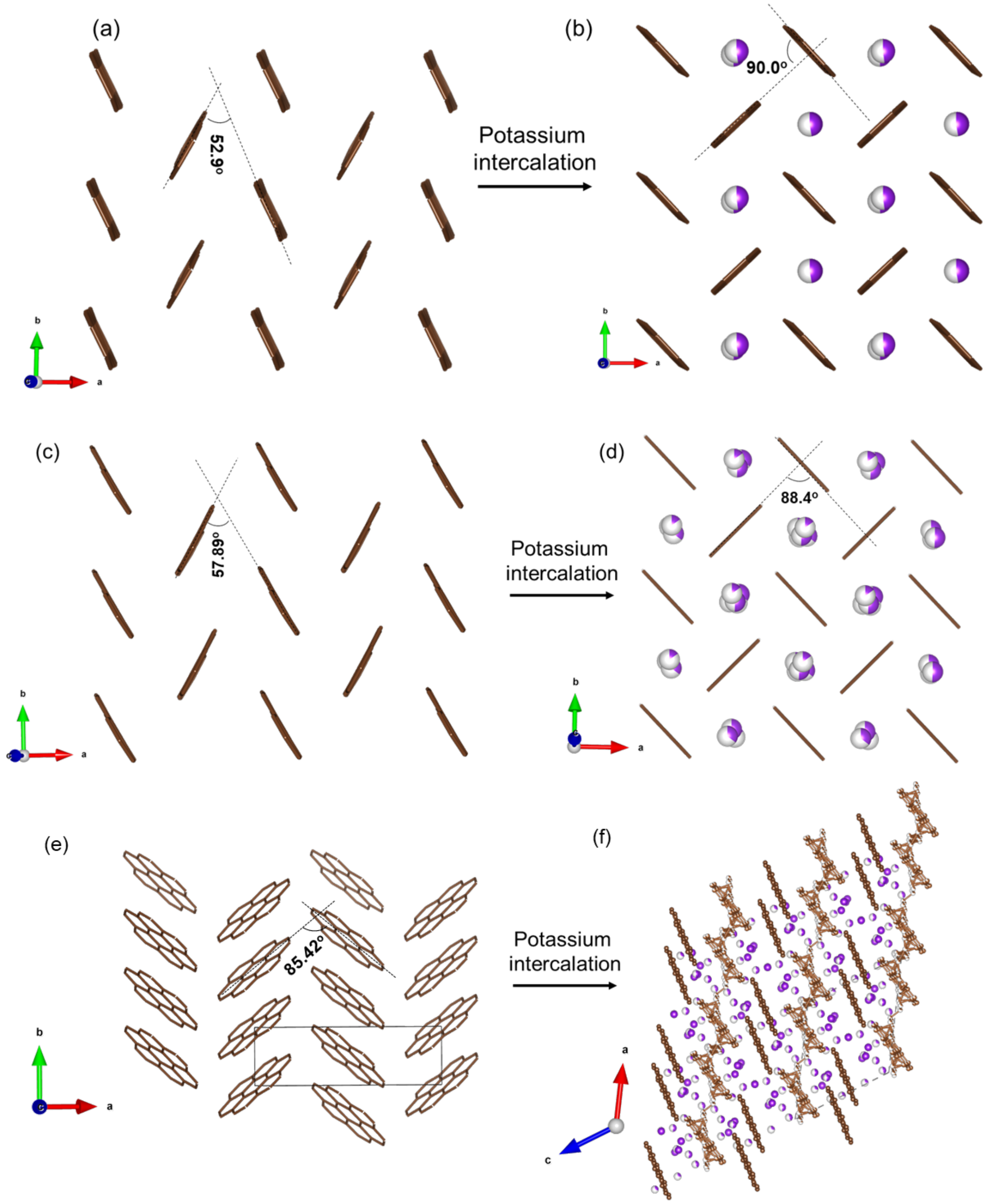

Figure S18. (a) Herringbone array in pristine pentacene[24], with an intermolecular angle of 52.9°. (b) Reorientation of the herringbone array, with an increase in the intermolecular angle to 90.0°, in order to create K sites in K_2pentacene.[25] (c) Herringbone array in pristine picene[26], with an intermolecular angle of 57.89°. (d) Reorientation of the herringbone array, with an increase in the intermolecular angle to 88.4°, in order to create K sites in K_2pentacene.[25] (e) Herringbone array in pristine coronene[6], with an intermolecular angle of 85.42°. (f) Extensive reorientation in the coronene arrangement after K^+ ions intercalation (inter-molecular angles shown in Figure 6a). While K_2pentacene and K_2picene maintains the herringbone packing after K^+ ions intercalation, in coronene the alternate layers realign almost in the same direction. The purple balls represent the K atoms.

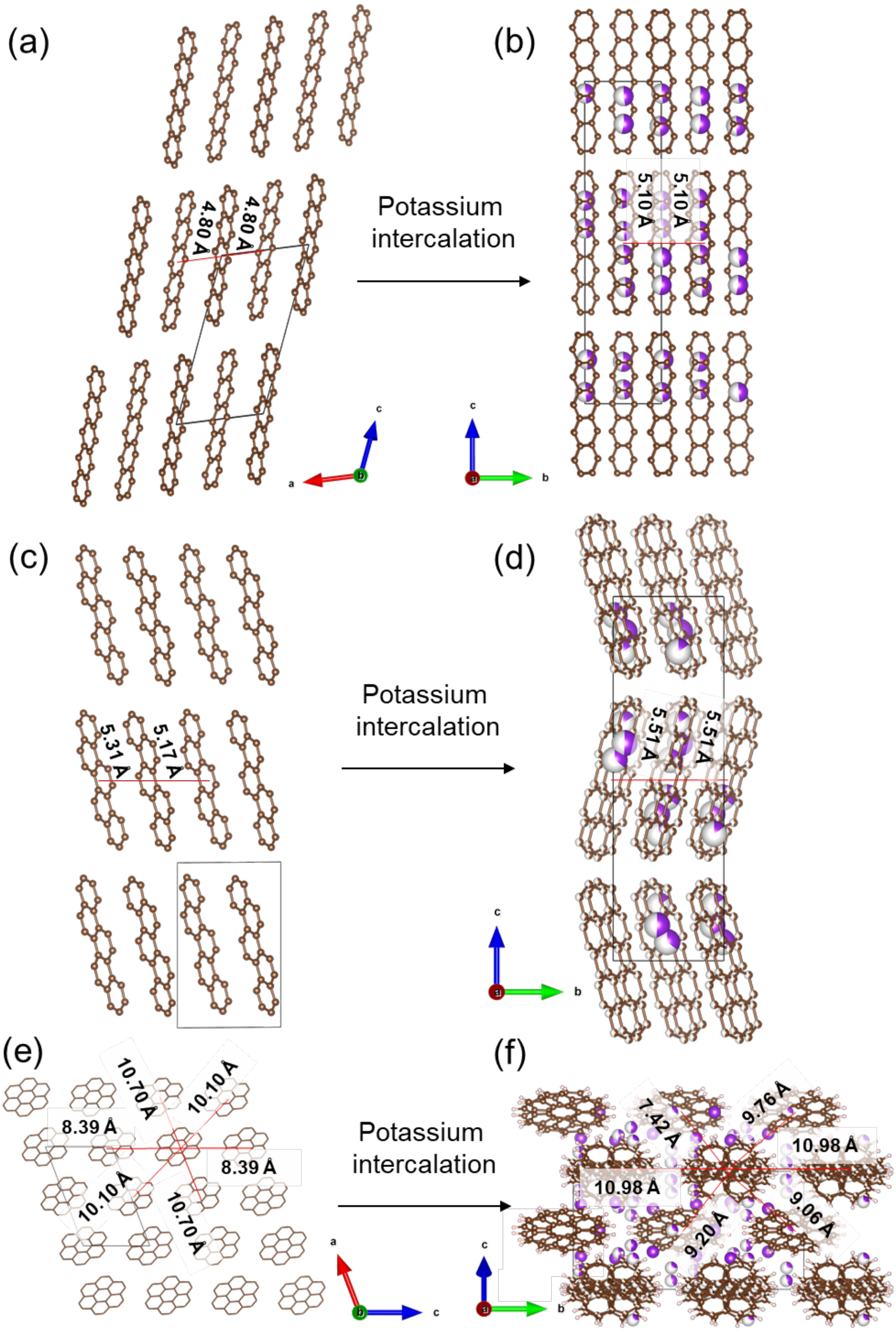

Figure S19. Packing of chains in pristine pentacene (a)[24], K_2pentacene (b)[25], pristine picene[26] (c) and K_2picene[25] (d), coronene[6] (e) and $K_{3.03(3)}$coronene (f). Due to linear nature of picene and pentacene, the chains are packed with two neighbouring chains instead of six as seen for coronene, which is disk shaped. The close packing amongst the chains retains in picene,

pentacene and coronene, although their spacing and orientation changes with K^+ ions intercalation.

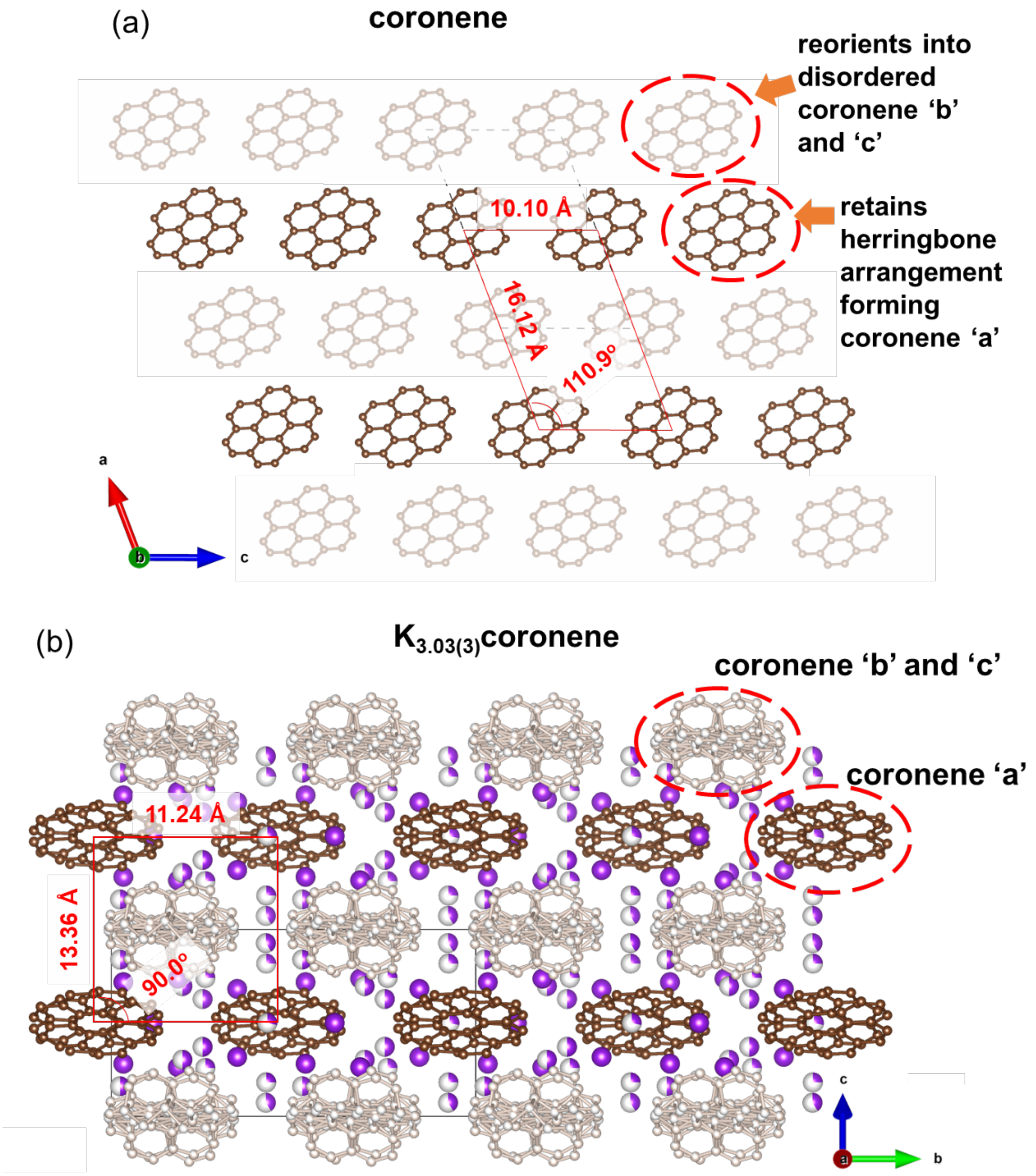

Figure S20. (a) Structure of pristine coronene and (b) $K_{3.03(3)}$coronene. The shaded coronene chains in (a) reorient in order to accommodate the K^+ ions, forming disordered coronene 'b' and 'c', while the other coronene chains undergo a minor re-orientation, but retain their herringbone arrangement forming coronene 'a', with the most significant change being the increased spacing between coronene molecules along the chain. The box here in red is representative of the coronene motifs (unshaded molecules) which remains after the K^+ ions (purple balls) incorporation, with alternative short and large coronene – coronene distances (the box does not represent the unit cell, the unit cell is given by the grey dashed lines).

Section S7. MAS-NMR and magnetization data.

^{13}C cross-polarization (CP) magic angle spinning (MAS) NMR measurements were performed on a Bruker Avance III 500 MHz spectrometer using a standard 4 mm CP-MAS probe. Experiments were carried out with CP-MAS zirconium rotors with 4 mm radius.

^{13}C CP MAS NMR spectra were collected at room temperature, at a spinning frequency of 10 kHz. The 90° pulse length was 4.4 μs. Typically, the data was collected with 51200 scans, a 1.6 ms CP pulse and a recycle delay of 4 s. Adamantane signal at 38.42 ppm relative to the ^{13}C signal of liquid tetra-methyl-silane (TMS) was used as the reference of ^{13}C chemical shifts. All shifts reported here are given relative to TMS standard.

Proton T_1 saturation recovery with CP detection was recorded using the same parameters as for ^{13}C measurements, with the number of scans reduced to 6144.

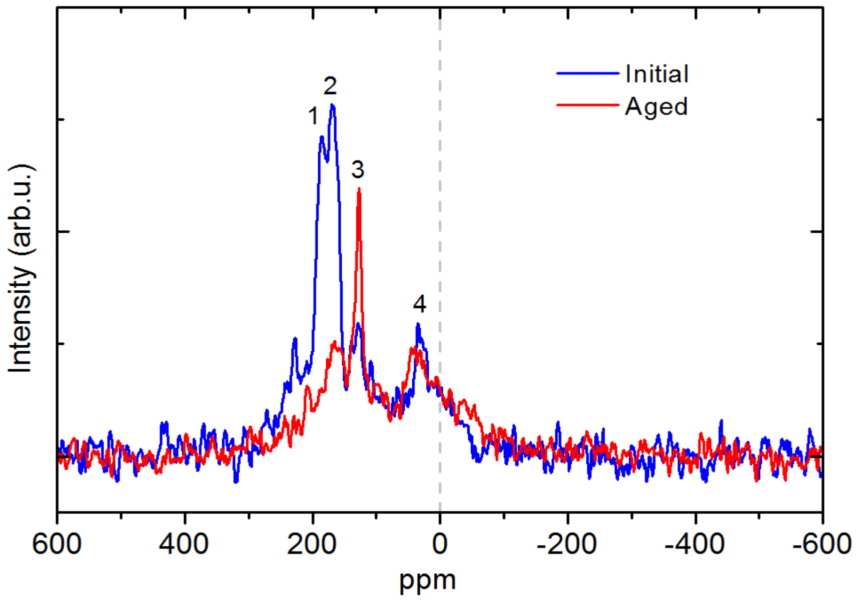

Figure S21. ^{13}C CP MAS NMR spectra of K_3coronene (K_3cor-1) sample. The data represented by the blue line is the initial measurement taken immediately after loading the sample into CP-MAS zirconium rotor. The data represented by the red line shows the measured spectrum, after the sample was kept in the rotor sample holder outside the glovebox for about 2 weeks. The rotor is sealed with caps that may not be completely air-tight so that slow degradation of air-sensitive sample takes place.

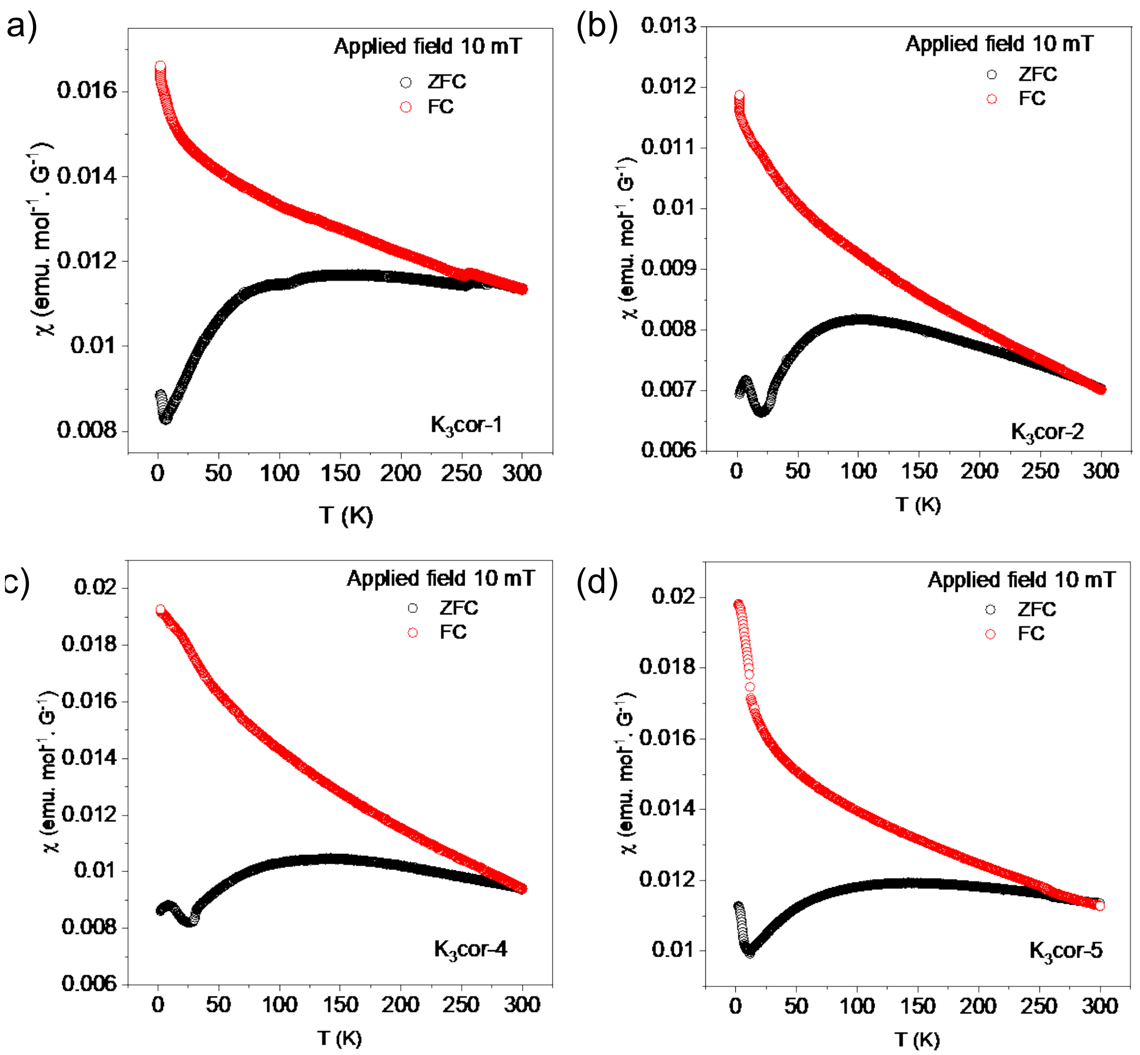

Figure S22. Temperature dependent magnetization for (a) K_3cor-1, (b) K_3cor-2, (c) K_3cor-4 and (d) K_3cor-5, under an applied field of 10 mT with zero field cooled (ZFC) and field cooled (FC) protocols. Temperature dependent ZFC-FC do not show any signature of superconductivity in K_3coronene in all the batches measured.

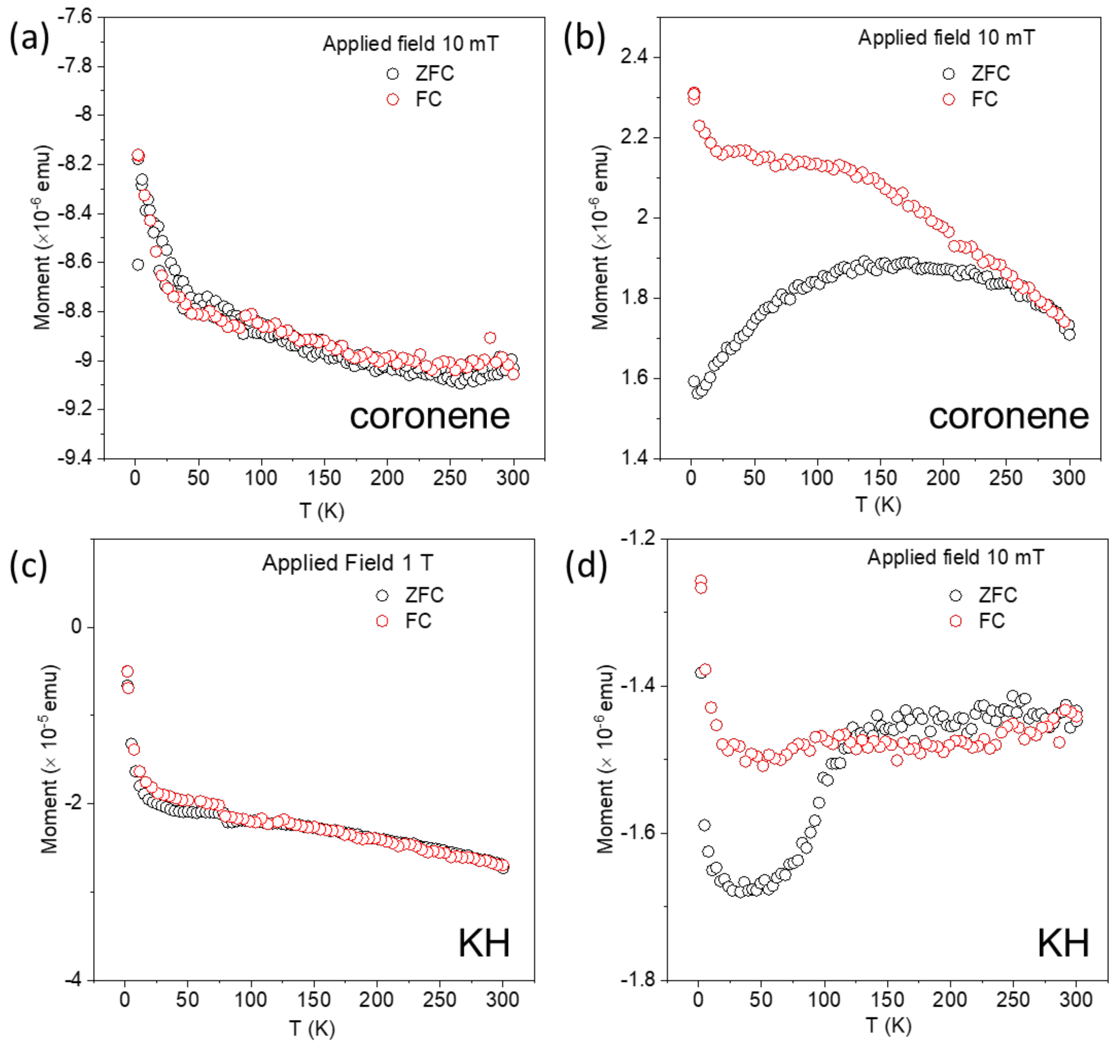

Figure S23. Temperature dependence of magnetization for as used coronene (a) before and (b) after being subjected to same temperature profile as K_3coronene. Temperature dependence of magnetization for as-used KH (c) before and (d) after being subjected to same temperature profile as K_3coronene. Coronene after subjecting to the same temperature profile of K_3coronene shows positive magnetic moment with divergent ZFC-FC temperature dependence similar to K_3coronene.

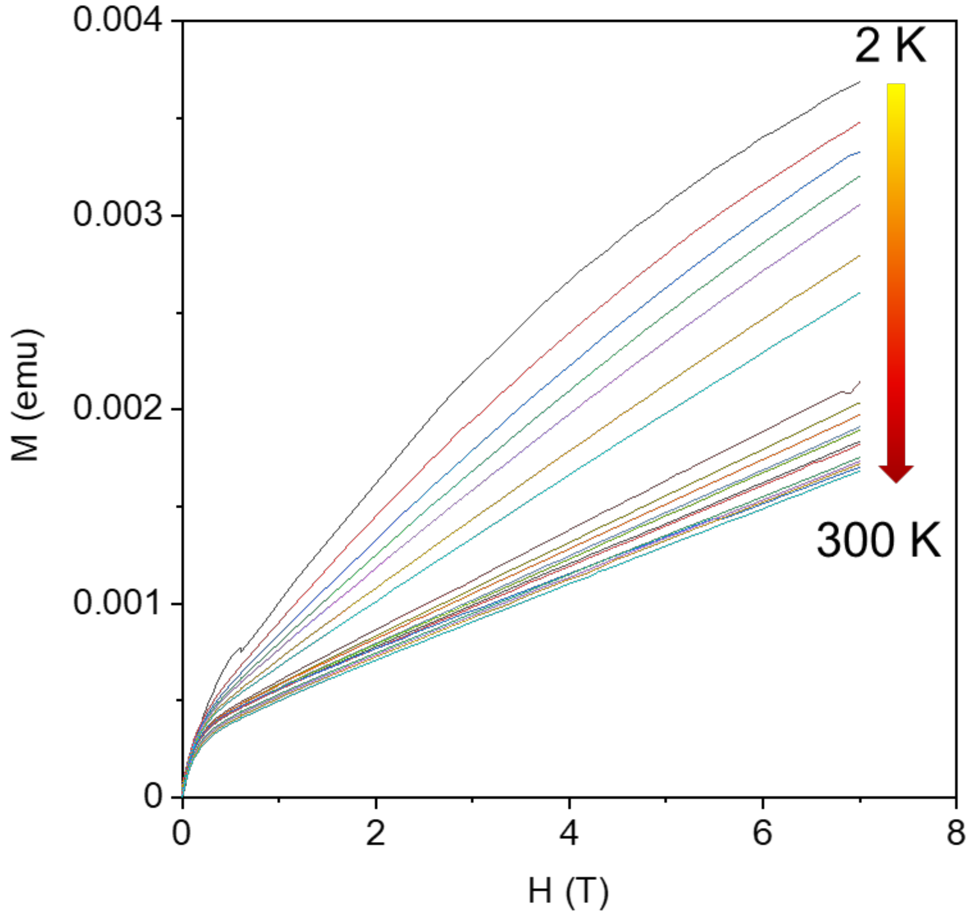

Figure S24. M(H) isotherms of K_3cor-1 in the applied field of 0 – 7 T

Table S5. Parameters from modified Curie-Weiss fit (10 – 300 K) of K_3coronene across different batches. Numbers in parentheses are errors in final digit.

**Measurement (MPMS-3)**	**K_3cor-1**	**K_3cor-2**	**K_3cor-4**	**K_3cor-5**
C (emu K mol^{-1} Oe^{-1})	0.0128(3)	0.0304(2)	0.0185(2)	0.0164(5)
θ_{CW} (K)	-3.0(3)	-5.2(1)	-9.2(2)	-1.7(27)
μ_{eff} (μ_B)	0.319(4)	0.492(2)	0.384(2)	0.361(6)
χ_0 (emu mol^{-1} Oe^{-1})	15.60(4) × 10^{-4}	4.34(1) × 10^{-4}	8.16(1) × 10^{-4}	9.28(56) × 10^{-4}
Number of unpaired electrons	0.05	0.11	0.07	0.06

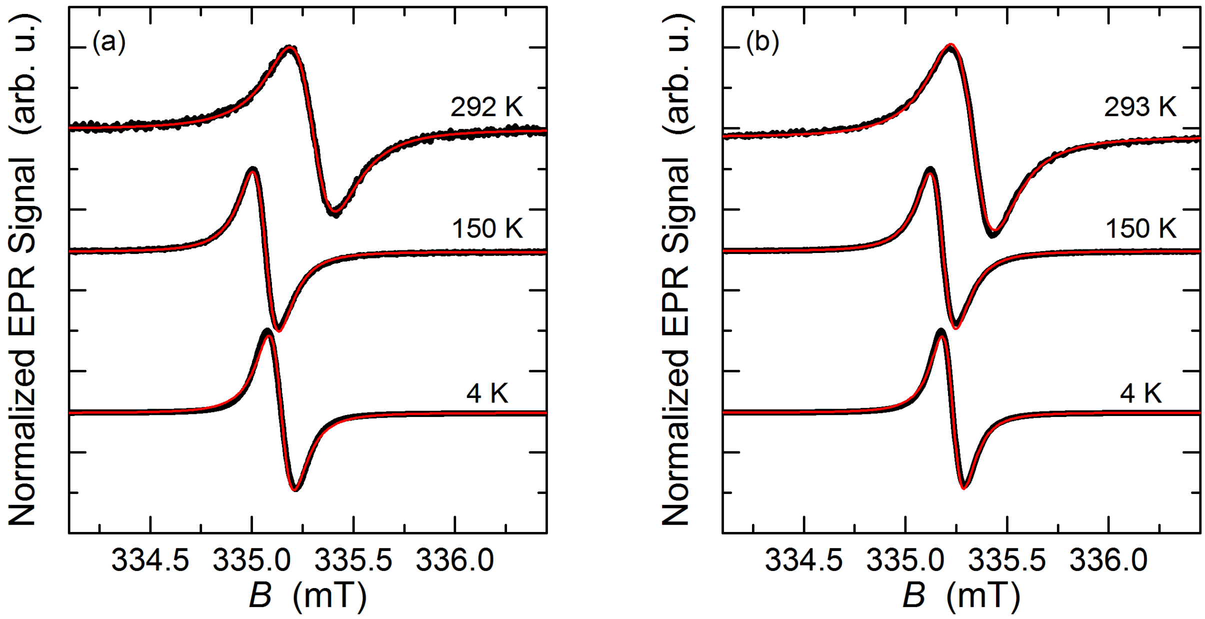

Figure S25. EPR spectra of $K_3C_{24}H_{12}$ (black lines) for two different samples, labelled K_3cor-3 (a) and K_3cor-2 (b), measured at three characteristic temperatures. The solid red line is a spectral fit to a single Lorentzian lineshape.

Section S8. DFT calculations.

To gain some additional information on the electronic structure, we first carried out DFT calculations for coronene molecules with a formal charge 1− and 3− (Figure S26). The corresponding molecular orbital energy levels are shown in Figure S26a where HOMO-LUMO gap values are 0.9 and 1.1 eV for $C_{24}H_{12}^{-}$ and $C_{24}H_{12}^{3-}$ respectively. These values are generally consistent with the literature.[2, 3, 5-10, 23-26] Calculated spin density for the singly occupied molecular orbital (SOMO) is distributed particularly at the contour of the planar molecules (Figure S26b). The main difference between $C_{24}H_{12}^{-}$ and $C_{24}H_{12}^{3-}$ anions is the loss of the mirror plane for the latter case. The shape of SOMO implies that the strongest direct exchange interaction between nearest neighbouring charged coronene molecules will be for their π-π stacking arrangement.

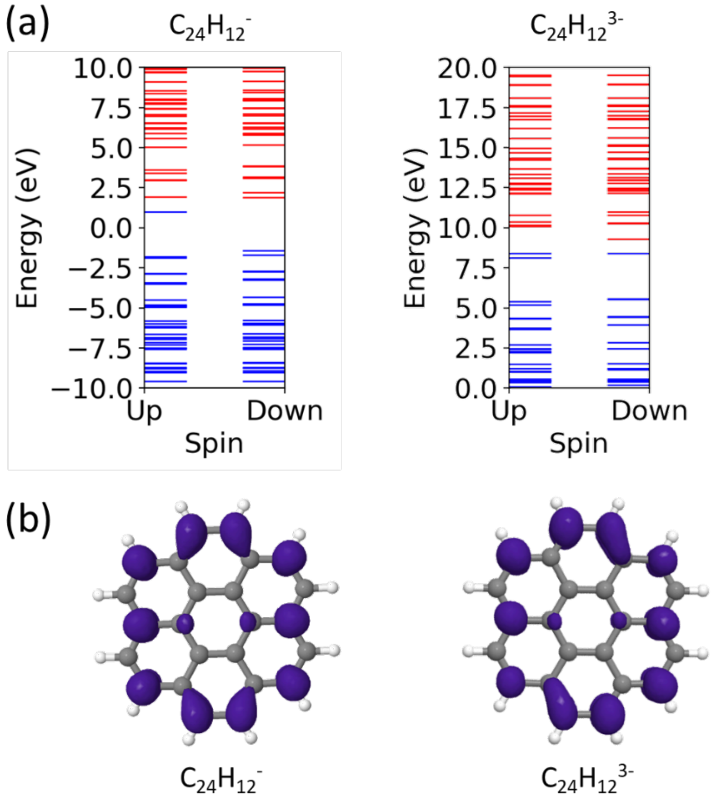

Figure S26. DFT-calculated (a) energy levels and (b) spin distribution of charged coronene $C_{24}H_{12}^{-}$ and $C_{24}H_{12}^{3-}$. In (a), red and blue lines represent unoccupied and occupied states at each energy. In (b), grey and white balls represent carbon and hydrogen atoms. Purple cloud shows spin distribution isosurface with a cutoff value set at 0.002 e/A^3.

In order to calculate the exchange coupling between pairs of charged (2− and 6−) coronene molecules, Broken-Symmetry (BS) DFT calculations were performed for paired-coronene

structures which are structure-optimized at the triplet state. Calculated exchange coupling, the difference between the energy of the triplet and the singlet states ($E_{triplet}$-$E_{singlet}$), result in small values of 0.03 meV and 0.05 meV for 2– and 6–, where the distance between the 2 coronene molecules were 5.3 Å and 4.7 Å, respectively. Note that these calculations were performed for pairs of parallel molecules. The calculated exchange suggests exchange coupling strength <1 K, which does not explain the large missing magnetic moment in our experiments. Thus, we next carried out BS-DFT for shorter intermolecular distances (2 Å and 4 Å) with 6– charge and varying geometry shift (0-6 Å) of one coronene molecule along the x-axis (along the zig-zag edge), without structure optimization in order to avoid unintended geometry shift. These two intermolecular distances were chosen to simulate coronene pairs found along the chain '1' and chain '2' (Figure S13).

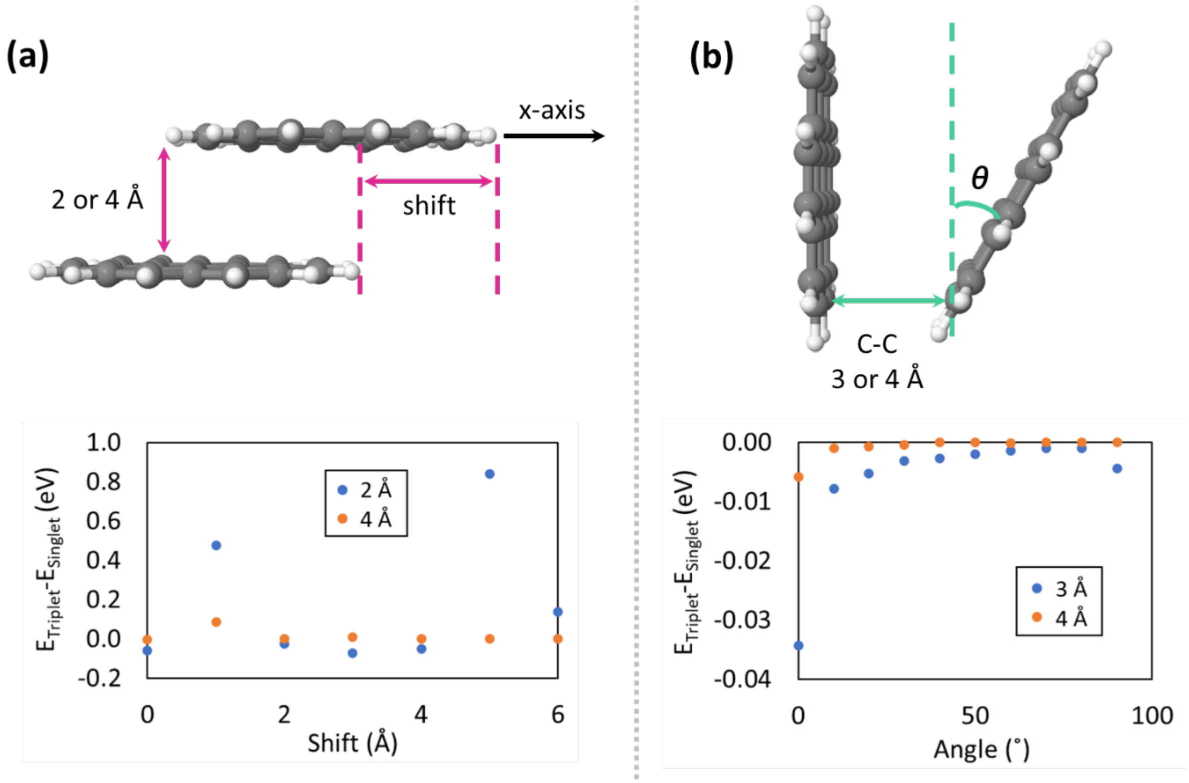

Figure S27. Calculated exchange overlap ($E_{triplet}$-$E_{singlet}$) of 2 coronene radicals with total charge 6–. (a) Top: schematic diagram of the definition of the shift along x-axis, Bottom: horizontal slide along x-axis keeping the intermolecular distance of 2 or 4 Å. (b) Top: schematic diagram of the definition of tilt angle θ, Bottom: different dihedral angles θ keeping the shortest intermolecular C-C distance at 3 or 4 Å.

Exchange coupling shown in Figure S27a is understandably the largest for the shortest intermolecular distance due to the larger orbital overlap. The value of $E_{triplet}$-$E_{singlet}$ is

significantly modified upon the relative horizontal molecular shift, because the orbital overlap with the two molecules, whose molecular orbital is mainly distributed at the coronene contour, varies with the shift. Experimentally observed structure (2 Å intermolecular distance and 2-4 Å shift) has -0.005~-0.025 eV (12-300 K in the temperature scale) of exchange coupling. The strength of these exchange couplings may thus indeed suggest the formation of singlets on nearest neighbouring coronene pairs.

Next, we calculated the exchange overlap ($E_{triplet}$-$E_{Singlet}$) of 2 coronene radicals with total charge 6− for different relative tilt angles θ (Figure S27b). As anticipated, the exchange overlap decays quickly with θ.

Having all molecular orbitals known we can next calculate EPR parameters, i.e., g-factor values and the hyperfine couplings to all carbons and hydrogens of coronene molecules. The isotropic part of g-factors is 2.00244 and 2.00224 for $C_{24}H_{12}^{-}$ and $C_{24}H_{12}^{3-}$, respectively. The minute deviation from the free electron value of 2.0023 in this case is not surprising as light elements carbon and hydrogen have negligibly small spin-orbit coupling. The loss of mirror plane in 3− charged molecule leads to a slightly larger g-factor anisotropy, e.g., g_y-g_x=9×10^{-4} compared to 6×10^{-4} for the 1− charged coronene. Isotropic hyperfine coupling parameters (A_{iso}) for the individual atomic sites are summarized in Tables S5 and S6. The strongest coupling to carbon C(11)/C(26) and C(14)/C(29) (see Table S5 and S6 for atom labels) amounts to 14.46 MHz and 14.24 MHz for $C_{24}H_{12}^{-}$ and $C_{24}H_{12}^{3-}$, respectively. Hyperfine coupling to hydrogens is also not negligible as the largest to hydrogen H(10)/H(25) and H(17)/H(32) amounts to 9.59 MHz and 10.21 MHz for $C_{24}H_{12}^{-}$ and $C_{24}H_{12}^{3-}$, respectively. The strong hyperfine coupling in these carbon and hydrogen atoms is not surprising as they are located at the positions of the largest SOMO spin density.

Table S6. Summary of EPR isotropic hyperfine coupling parameters, A_{iso}, and associated atom labels for $C_{24}H_{12}^{-}$. In the molecular model, grey/white-numbering and white/black-numbering balls represent carbon and hydrogen atoms, respectively. Purple cloud shows spin distribution isosurface with a cutoff value set at 0.002 e/A^3. In the table, shaded lines indicate carbon atoms in the molecular model.

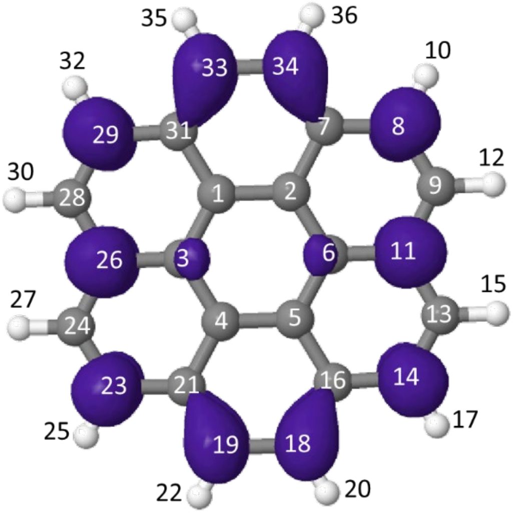

Label	A_{iso} (MHz)	Label	A_{iso} (MHz)
1	-0.9613	10	-9.5868
2	-0.9561	17	-9.5412
4	-0.9560	25	-9.5869
5	-0.9611	32	-9.5411
3	-2.8147	11	14.4562
6	-2.8146	26	14.4560
7	-6.9187	12	3.2611
16	-6.8325	15	3.2557
21	-6.9189	27	3.2610
31	-6.8324	30	3.2555
8	12.1005	18	5.2209
14	12.0244	19	5.3245
23	12.1006	33	5.2212
29	12.0244	34	5.3247
9	-13.0738	20	-7.5828
13	-13.0476	22	-7.6331
24	-13.0736	35	-7.5830
28	-13.0477	36	-7.6332

Table S7. Summary of EPR isotropic hyperfine coupling parameters and atom labels for $C_{24}H_{12}^{3-}$. In the molecular model, grey/white-numbering and white/black-numbering balls represent carbon and hydrogen atoms, respectively. Purple cloud shows spin distribution isosurface with a cutoff value set at 0.002 e/A^3. In the table, shaded lines indicate carbon atoms in the molecular model.

Label	A_{iso} (MHz)	Label	A_{iso} (MHz)
1	-0.5541	13	-11.1457
5	-0.5544	28	-11.1458
2	-1.2070	14	14.2421
4	-1.2067	29	14.2426
3	-1.9843	15	1.5439
6	-1.9841	30	1.5438
7	-2.4410	16	-11.6061
21	-2.4412	31	-11.6062
8	6.2393	17	-10.2067
23	6.2395	32	-10.2066

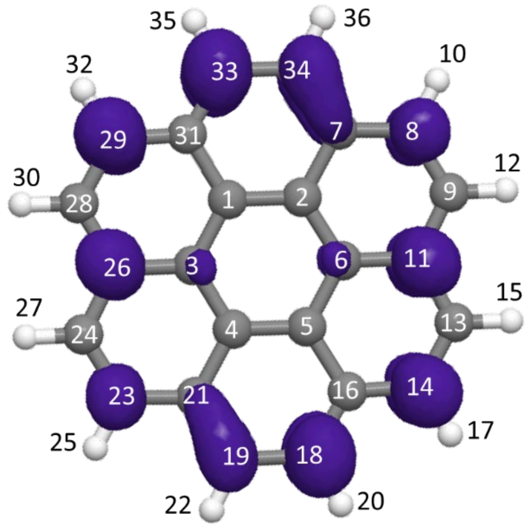

9	-9.0055	18	11.2280
24	-9.0053	33	11.2280
10	-5.6256	19	1.1295
25	-5.6257	34	1.1296
11	11.1904	20	-10.2773
26	11.1905	35	-10.2772
12	1.4254	22	-5.6030
27	1.4251	36	-5.6031

REFERENCES

1. A. G. De La Torre, S. Bruque and M. A. G. Aranda, *J. Appl. Crystallogr.*, 2001, **34**, 196-202.
2. T. Sterling and J. J. Irwin, *J. Chem. Inf. Model.*, 2015, **55**, 2324-2337.
3. J. J. P. Stewart, *J. Mol. Model.*, 2007, **13**, 1173-1213.
4. J. H. Ward Jr, *J. Am. Stat. Assoc.*, 1963, **58**, 236-244.
5. T. F. Willems, C. H. Rycroft, M. Kazi, J. C. Meza and M. Haranczyk, *Microporous Mesoporous Mater.*, 2012, **149**, 134-141.
6. E. Fawcett, J. Trotter and J. M. Robertson, *Proc. R. Soc. London, Ser. A*, 1966, **289**, 366-376.
7. G. Kresse and J. Furthmüller, *Phys. Rev. B*, 1996, **54**, 11169-11186.
8. J. Klimeš, D. R. Bowler and A. Michaelides, *Phys. Rev. B*, 2011, **83**, 195131.
9. G. Kresse and D. Joubert, *Phys. Rev. B*, 1999, **59**, 1758-1775.
10. A. Coelho, *J. Appl. Crystallogr.*, 2000, **33**, 899-908.
11. F. Neese, *WIREs Comput. Mol. Sci.*, 2012, **2**, 73-78.
12. F. Neese, *WIREs Comput. Mol. Sci.*, 2022, **12**, e1606.
13. A. D. Becke, *J. Chem. Phys.*, 1993, **98**, 5648-5652.
14. T. Clark, J. Chandrasekhar, G. W. Spitznagel and P. V. R. Schleyer, *J. Comput. Chem.*, 1983, **4**, 294-301.
15. P. C. Hariharan and J. A. Pople, *Theor. Chim. Acta*, 1973, **28**, 213-222.
16. R. Ditchfield, W. J. Hehre and J. A. Pople, *J. Chem. Phys.*, 1971, **54**, 724-728.
17. W. J. Hehre, R. Ditchfield and J. A. Pople, *J. Chem. Phys.*, 1972, **56**, 2257-2261.
18. B. P. Pritchard, D. Altarawy, B. Didier, T. D. Gibson and T. L. Windus, *J. Chem. Inf. Model.*, 2019, **59**, 4814-4820.
19. D. Feller, *J. Comput. Chem.*, 1996, **17**, 1571-1586.
20. K. L. Schuchardt, B. T. Didier, T. Elsethagen, L. Sun, V. Gurumoorthi, J. Chase, J. Li and T. L. Windus, *J. Chem. Inf. Model.*, 2007, **47**, 1045-1052.
21. V. Barone, in *Recent Advances in Density Functional Methods*, World Scientific, 1995, vol. Volume 1, pp. 287-334.

22. C. F. Macrae, I. Sovago, S. J. Cottrell, P. T. A. Galek, P. McCabe, E. Pidcock, M. Platings, G. P. Shields, J. S. Stevens, M. Towler and P. A. Wood, *J. Appl. Crystallogr.*, 2020, **53**, 226-235.
23. C. R. Groom, I. J. Bruno, M. P. Lightfoot and S. C. Ward, *Acta Crystallogr., Sect. B*, 2016, **72**, 171-179.
24. G. R. Desiraju and A. Gavezzotti, *Acta Crystallogr., Sect. B*, 1989, **45**, 473-482.
25. F. D. Romero, M. J. Pitcher, C. I. Hiley, G. F. S. Whitehead, S. Kar, A. Y. Ganin, D. Antypov, C. Collins, M. S. Dyer, G. Klupp, R. H. Colman, K. Prassides and M. J. Rosseinsky, *Nat. Chem.*, 2017, **9**, 644-652.
26. A. De, R. Ghosh, S. Roychowdhury and P. Roychowdhury, *Acta Crystallogr., Sect. C*, 1985, **41**, 907-909.
